# The distributional consequences of social distancing on poverty and labour income inequality in Latin America and the Caribbean

**DOI:** 10.1007/s00148-021-00854-1

**Published:** 2021-07-28

**Authors:** Isaure Delaporte, Julia Escobar, Werner Peña

**Affiliations:** 1 University of St Andrews, St Andrews, Fife, KY16 9AL Scotland UK; 2 Inter-American Development Bank, Washington, DC USA; 3 School of Economics, University of Kent, Canterbury, CT2 7FS Kent UK

**Keywords:** COVID-19, Social distancing, Compliance, Employment, Poverty, Labour income inequality, D33, E24, J21, J31

## Abstract

This paper estimates the potential distributional consequences of the first phase of the COVID-19 lockdowns on poverty and labour income inequality in 20 Latin American and Caribbean (LAC) countries. We estimate the share of individuals that are potentially able to remain active under the lockdown by taking into account individuals’ teleworking capacity but also whether their occupation is affected by legal workplace closures or mobility restrictions. Furthermore, we compare the shares under the formal (de jure) lockdown policies assuming perfect compliance with the shares under de facto lockdowns where there is some degree of non-compliance. We then estimate individuals’ potential labour income losses and examine changes in poverty and labour income inequality. We find an increase in poverty and labour income inequality in most of the LAC countries due to social distancing; however, the observed changes are lower under de facto lockdowns, revealing the potential role of non-compliance as a coping strategy during the lockdowns. Social distancing measures have led to an increase in inequality both between and within countries. Lastly, we show that most of the dispersion in the labour income loss across countries is explained by the sectoral/occupational employment structure of the economies.

## Introduction

To prevent the spread of the COVID-19 pandemic, governments around the world have imposed social distancing measures which have had an asymmetric effect on the labour market. While some sectors have been considered essential and thus essential workers have continued to go to work and to receive their wages, other sectors have had to close or have been affected by mobility restrictions because of the high risk of transmission of the virus that these activities entail. Among the individuals that have been asked to stay at home, some have been able to remain active due to the task content of their occupations (Dingel and Neiman 2020; Delaporte and Peña 2020; Gottlieb et al. 2021; Hatayama et al. 2020; Barbieri et al. 2020; Béland et al. 2020; Hensvik et al. 2020; Holgersen et al. 2020; Yasenov 2020), while others have not been able to work from home and have experienced wage losses. Therefore, the effect of social distancing policies could be significant in terms of labour income inequality and poverty rates.

A rapidly growing literature analyses the distributional effects of the lockdown policies on poverty and inequality (Palomino et al. 2020; Perugini and Vladisavljevic 2020; Brunori et al. 2020; Bonacini et al. 2021b; Duman 2020; Bonavida Foschiatti and Gasparini 2020; Lustig et al. 2020; Leone 2020; Botha et al. 2021).^1^ Among the existing studies, our paper is closely related to Palomino et al. (2020) and Duman (2020). Palomino et al. (2020) evaluate the capacity of individuals in Europe to work under a lockdown based on a Lockdown Working Ability index that considers individuals’ teleworking capacity and whether their occupation is essential or closed. The authors rely on microsimulation techniques to examine the changes in poverty and inequality under different lockdown intensity and duration. They find an increase in both poverty and inequality in all European countries. Duman (2020) follows a similar methodology to examine the case of Turkey and finds that the overall negative distributional effects of the lockdown become more substantial with duration.

We contribute to the existing literature in a number of ways. First, this paper evaluates the potential distributional consequences of social distancing on poverty and labour income inequality in 20 Latin American and Caribbean (LAC) countries. To do so, this study relies on two data sources. First, we gather detailed information from national laws, decrees and press releases on the strictness and the duration of the first phase of the lockdown in each LAC country.^2^ The strictness of the lockdown measures is defined in terms of both workplace closures and mobility restrictions. Furthermore, when countries have adopted a regional approach for the lockdowns, we identify the measures at the regional level in order to capture the heterogeneity in the lockdown intensity and duration across regions within countries. Second, we use rich household surveys harmonised by the Inter-American Development Bank (IADB). The surveys used for this study cover 20 countries, including one North American country, ten South American countries, five Central American countries and four Caribbean countries.^3^ The surveys contain harmonised individual-level data on demographic, labour and income conditions. More specifically, we have information on workers’ occupations, economic activities and labour income.

Second, this paper conducts a novel ex-post assessment of the potential implications of the COVID-19 lockdowns on poverty and inequality, thus departing from the strategy of simulating ex-ante impacts under different scenarios of lockdown. To carry out the analysis, we partly follow Palomino et al. (2020) and Duman (2020) and construct the Lockdown Working Ability (LWA) index that represents the capacity of individuals to remain active under the first phase of the lockdown given their teleworkability index, i.e. the feasibility to work from home, but also whether their economic activity/occupation is affected by legal workplace closures or mobility restrictions. In particular, we assume that individuals who work in open sectors remain active while those who work in closed sectors do not. The remaining activities, which are neither open nor closed, are affected by mobility restrictions. As a result, the workers in these sectors are considered as active depending on their capacity to perform their job from home. However, we depart from existing studies on the following aspects. First, our LWA measure is based on a country-specific lockdown policy. In particular, the classification of sectors as essential or closed is unique to each country. Furthermore, when countries have adopted a regional approach, we classify the sectors as essential or closed according to the measures implemented in each region.^4^ Similarly, the duration of the first phase of the lockdown varies across countries and regions.^5^

Third, this paper contributes to the existing literature by comparing the formal (de jure) lockdown policies when perfect compliance is assumed with de facto lockdowns when there is some degree of non-compliance. The difference is likely to be important in the context of LAC countries for several reasons (Yeyati and Sartorio 2020a; Yeyati and Valdés 2020). First, the region is characterised by a high rate of informality. Besides, a large share of the population lives in poverty and in overcrowded habitats. Furthermore, governments’ income support programmes are often limited (Busso et al. 2020). As a result, people’s capability and willingness to comply with restrictive policies is likely to differ across places and over time (Galasso et al. 2020; Yeyati and Sartorio 2020a). In addition, non-compliance is relevant from an economic point of view as it allows us to uncover the role of a potential coping strategy that has received little attention. Indeed, non-compliance can be a mechanism to smooth labour income losses related to lockdowns. To take this into consideration, we modify the LWA measure to allow for some degree of non-compliance. More specifically, we assume that, for closed and restricted activities, the proportion of individuals that remain active depends additionally on the level of non-compliance at the regional level in each country. To estimate the degree of non-compliance, we follow Yeyati and Sartorio (2020a) and use the Oxford Stringency Index (OSI), which constitutes a continuous measure of the intensity of the formal lockdown policies over time, as well as Google workplace mobility data to proxy for de facto lockdowns. By normalizing and taking the difference between the two measures, we obtain a proxy of compliance over time at the regional level in each country.

Once individuals that are able to work have been identified, the next step is to calculate individuals’ potential labour income losses due to social distancing given that the duration of the first phase of the lockdown varies across countries. We examine how the mean loss labour income rate varies across occupations, economic activities and specific population groups within countries. Furthermore, we compare the mean loss labour income rate under de jure and de facto lockdowns. Then, by comparing the pre-lockdown situation with the situation at the end of the initial phase of the lockdown, we measure the changes in poverty and labour income inequality across countries. We follow Palomino et al. (2020) and use a series of measures to illustrate these changes. First, for our analysis on poverty, we compute for each country the Lockdown Incidence Curve (LIC), which represents the relative change in the labour income of individuals ordered by percentiles. In addition, we compute the Foster-Greer-Thorbecke indices to estimate changes in the share of workers living with a labour income below the international poverty line as well as changes in the median poverty gap and in the severity of poverty.^6^ Second, to calculate the changes in labour income inequality, we use the Gini coefficient and the Mean Logarithmic Deviation (MLD) index. While the first measure is traditionally used to study inequality, the second measure allows us to decompose overall inequality into a between-group and a within-group component (Bourguignon 1979). We compute all the measures under perfect and imperfect compliance.

Our results show considerable variation across countries in the share of individuals potentially able to remain active under the first phase of the lockdown. This share also varies significantly within countries across occupations, economic activities and specific population groups. Our results on the potential labour income losses show different effects across countries. For instance, in Argentina, the Bahamas, Barbados, Brazil, Colombia, the Dominican Republic and El Salvador, the bottom percentiles are the most affected. By contrast, in Belize, Chile, Costa Rica, Ecuador, Jamaica, Paraguay and Uruguay, all parts of the labour income distribution suffer relatively similar losses. Across occupations, craft and related trades workers suffer the largest losses. Across economic activities, workers in the construction sector experience significant labour income losses as well. Further analysis suggests that the potential losses do not differ significantly by gender and level of education. However, informal workers have higher potential labour income losses than formal workers. Finally, introducing non-compliance attenuates the labour income losses across the income distribution.

Concerning our analysis on poverty and inequality, we find an increase in the share of workers living in poverty in almost all countries. Under perfect compliance, the highest increase in the headcount poverty index is observed in Guatemala. We also find that labour income inequality increases for the Gini coefficient and the MLD in almost all countries. The largest increase in the Gini coefficient is observed in El Salvador, while the largest increase in the MLD index is in Brazil when assuming perfect compliance. Overall, the observed changes in poverty and inequality are lower under de facto compliance, highlighting the potential role of non-compliance in LAC countries as a coping strategy during the lockdowns.^7^ Lastly, we decompose overall inequality for the LAC region into a between-countries and a within-countries component and find that social distancing has led to an increase in inequality both between and within countries. These changes are reduced in magnitude under imperfect compliance, but the pattern remains the same: between-country inequality increases significantly more than within-country inequality.

There are several potential explanations for these observed changes in poverty and labour income inequality. The increases are in general larger in countries that have implemented stricter and longer lockdowns but also in countries that are characterised by a higher share of jobs that cannot be performed from home. To better understand differences across countries, we conduct a series of counterfactual exercises to disentangle two reasons for the dispersion in the labour income loss across countries, namely the stringency of the lockdown policy and, conditional on implementing a lockdown, the sectoral/occupational employment structure of the economy. More specifically, we borrow from Caselli (2005) the inter-percentile differential measure and compute it after applying to all countries a common lockdown policy.^8^ The results of these simulations show that on average 75% of the cross-country labour income loss dispersion in the LAC region is explained by the sectoral/occupational employment structure of the economies. This result highlights the importance of considering the sectoral/occupational employment structure of the economy when implementing lockdowns, as this is a key factor in determining the magnitude and dispersion of potential labour income losses.

The rest of the paper proceeds as follows. In Sect. 2, we present the data and estimate teleworking capacity before explaining how the LWA index is constructed. In Sect. 3, we explain the methodology applied to calculate the changes in poverty and labour income inequality and present the results for the distributional effects of de jure and de facto lockdowns in LAC countries. Lastly, Sect. 4 concludes.

## The capacity to work under COVID-19

In this section, we first present the individual-level data that is used for the analysis. We then estimate the feasibility to work from home before presenting the Lockdown Working Ability (LWA) index which captures individuals’ ability to work during the lockdown. We propose two measures of the LWA index: the first measure assumes perfect lockdown compliance and takes into account individuals’ teleworking capacity but also whether individuals’ occupation is affected by workplace closures and mobility restrictions, while the second measure allows additionally for some degree of non-compliance.

### Individual-level data

This study uses rich household surveys from the IADB covering 20 LAC countries: Argentina, the Bahamas, Barbados, Belize, Bolivia, Brazil, Chile, Colombia, Costa Rica, the Dominican Republic, Ecuador, El Salvador, Guatemala, Jamaica, Mexico, Nicaragua, Paraguay, Peru, Uruguay and Venezuela. For each country, we use the most recent harmonised survey that is available.^9^ Table 3 in the Appendix provides the name of the survey, the year and the number of individuals in the sample for each country. The surveys contain harmonised individual-level data on demographic, educational, labour, income and housing conditions. More specifically, we have information on workers’ occupations, economic activity and annual labour income.

It should be noted that the information collected for Argentina and the Bahamas is only representative of the urban areas. Therefore, the results for these two countries are not directly comparable to the ones for the other countries. Indeed, the feasibility to work from home is usually higher in urban compared to rural areas (Delaporte and Peña 2020). In the opposite, the share of individuals able to remain active under the lockdown is expected to be lower in urban areas. This is due to the fact that the virus is more easily spread in urban areas due to population density and as a result the lockdown measures are stricter in urban compared to rural areas.^10^

### The teleworkability index

A rapidly growing literature since the beginning of the pandemic has been focusing on estimating the feasibility to work from home for individuals across the world. The existing studies differ in their approach. The first study by Dingel and Neiman (2020) uses information about the task content of occupations in the US to estimate the share of jobs that can potentially be done from home. The authors use surveys from the Occupational Information Network (O*NET). Others have adopted the same approach and have relied on the O*NET data to estimate the capacity to work from home in varied national contexts (Mongey and Weinberg 2020; Béland et al. 2020; Yasenov 2020; Duman 2020; Gallacher and Hossain 2020). However, it has been argued that the task content of occupations may vary significantly across contexts and that US-based measures might not be the most representative for developing economies.

To address this concern, Gottlieb et al. (2021) rely on the World Bank’s Skills Toward Employability and Productivity (STEP) surveys which provide information about the task content of occupations in 10 developing economies. They find a lower share of jobs that can be performed at home in these 10 developing economies compared to when O*NET is used. Similarly, Hatayama et al. (2020) rely on the Surveys of Adult Skills of PIAAC, the STEP surveys and the Labor Market Panel Surveys (LMPS) to calculate the feasibility of working from home in 53 countries. Other studies focus on specific countries and have used a country-specific task content of occupations to calculate the share of teleworkability (Barbieri et al. 2020; Bonacini et al. 2021b; Holgersen et al. 2020). More recently, a few studies have been able to rely on data collected during the pandemic and have provided real-time measures of the capacity for individuals to work from home (Brynjolfsson et al. 2020; Hensvik et al. 2020; Leone 2020).

With respect to Latin American and Caribbean countries, since information on the task content of occupations is not available specifically for each country of our sample, we adopt the following approach. We construct our measure of teleworkability capturing the feasibility for each occupation to be performed from home by using information about the task content of occupations from the STEP surveys. More specifically, there are two LAC countries sampled in the STEP surveys: Bolivia and Colombia. Since the task content of occupations in these two countries is likely to be more representative of the task content of occupations in other LAC countries^11^ than if we were to use US-based measures, we use the information provided by these two countries for the 20 countries included in our sample. It should be noted that the STEP surveys are restricted in their geographical scope to urban areas only and were collected in 2012. Regarding the geographical coverage of the surveys, this might lead to an overestimation of the proportion of individuals able to work from home. However, it is important to note that the COVID-19 pandemic has accelerated the process of technological change, which in turn might have increased the teleworkability share of the LAC economies. Regarding the fact that the information was collected in 2012, it would constitute an issue only if the task content of occupations has changed dramatically over the last decade.

Following the methodology of Gottlieb et al. (2021), we classify workers as unable to work from home if they either do not use a computer at work, lift heavy objects, repair electronic equipment, operate heavy machinery or report that customer interaction is very important. Once workers have been classified accordingly, we can obtain the share of individuals that can work from home by country and occupation. We take the average of the share of individuals that are able to work from home in Bolivia and Colombia at the 2-digit ISCO level. We can then merge the average share obtained for all 2-digit ISCOs from Bolivia and Colombia using our own 2-digit ISCO variable in our individual-level data. In this respect, we construct a harmonised version of the 2-digit ISCO-08 categories, which was not available in the IADB surveys.^12^ We do so to gain in precision. Indeed, there is a lot of heterogeneity in the task content of the occupations within the 1-digit occupational categories. Therefore, estimating the teleworkability share at the 1-digit ISCO level would lead to biased results.

**Fig. 1:**
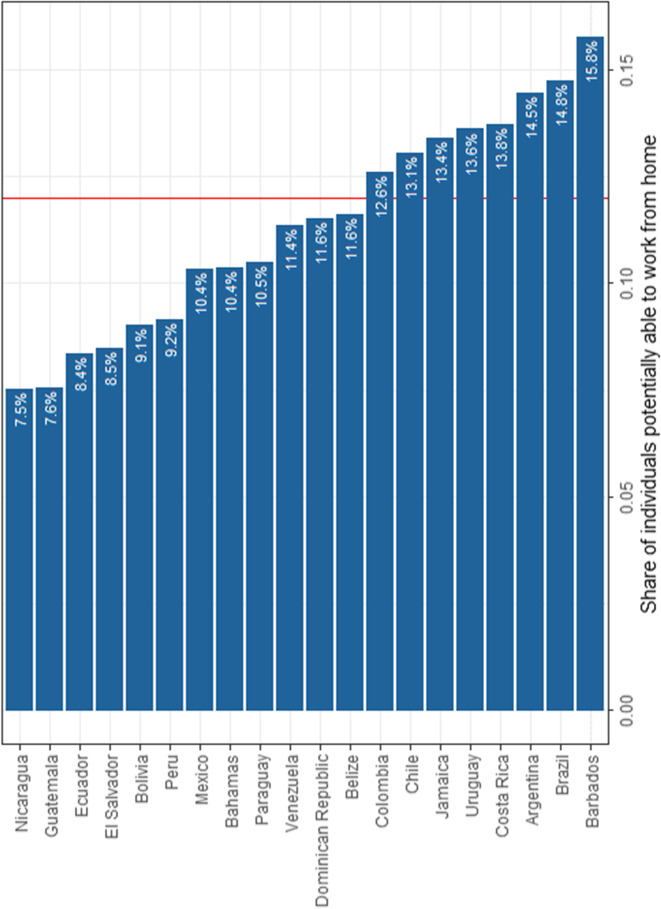
Share of individuals potentially able to work from home, by country. Source: Harmonised Household Surveys of Latin America and the Caribbean, authors’ own calculations. Notes: The average share of individuals able to work from home (represented by the vertical red line) is 12% for the entire LAC region. The LAC share was calculated as a weighted average of the population in all countries, excluding Argentina and the Bahamas, which are not representative at the national level

Once we have merged the average shares, the next step is to apply weights using the country-specific ISCO’s employment shares. By proceeding this way, we obtain a share of individuals able to work from home that varies across countries. This is due to the fact that countries have different sectoral/occupational employment structures. Figure 1 presents the shares of individuals potentially able to work from home by country. While the average share of individuals able to work from home is 12% for the entire LAC region, the proportion of individuals able to work from home varies across countries from 7.5 to 16%. The country with the lowest share of teleworkability in our sample is Nicaragua while the country with the highest share is Barbados.

We compare our teleworkability index with other measures. More specifically, we construct the teleworkability index following the approach of Dingel and Neiman (2020) using US-based measures. Overall, our measure indicates a lower estimated share of teleworkability, which confirms the presumption that O*NET-based measure overestimates the teleworking capacity of developing countries (Gottlieb et al. 2021; Hatayama et al. 2020). We also compare the shares that we obtain for specific countries with the shares obtained in existing studies using real-time data and conclude that our findings are in line with those of studies using real-time shares of people in homeworking. For instance, Leone (2020) finds virtually the same share for Brazil and Gottlieb et al. (2021) present evidence for Costa Rica, where 10.8% of urban workers worked remotely in the second quarter of 2020. The National Institute of Statistics of Chile reported a teleworking share of 22.1% in the month of April of 2020^13^ (Instituto Nacional de Estadística de Chile 2020), while IPSOS (2020) conducted a nationally representative urban survey in Peru and found that, in June 2020, 12% of the respondents were working from home. These statistics provide support to our findings.

We examine how the share of individuals able to work from home varies within countries across occupations (Appendix, Table 4). The results show that a larger proportion of workers are able to work from home among higher skilled occupations. For instance, the share of teleworkability is higher among clerical support workers (45% for the full sample), professionals (31% for the full sample), managers (29% for the full sample) and technicians and associate professionals (26% for the full sample). It is much lower for plant and machine operators and assemblers, as well as for agricultural workers and individuals in elementary occupations.

Across economic activities (Appendix, Table 5), the highest share of teleworkability is found in financial insurance and the real estate sector (31% for the full sample). It varies however considerably across countries, from 15% in the Bahamas to 39% in the Dominican Republic. Teleworking is also possible for a significant share of individuals in social and community services as well as in the electricity, gas and water sector (18% and 17% respectively for the full sample). However, as expected, individuals are much less likely to be able to work from home when they work in agriculture (0.007% for the full sample).

Lastly, we examine how the share of teleworkability differs across population groups (Appendix, Table 6). The results for the full sample indicate that men are less likely to be able to work from home compared to women (10% compared to 15%). A larger teleworkability share is found as well among individuals that have a higher level of education and that live in an urban area. Informal workers are more affected: only 7% of them are able to work from home compared to 18% of the formal workers. Lastly, individuals in the top of the labour income distribution have higher capacity to work from home compared to those in the bottom part (21% compared to only 7% of the individuals in the bottom quintile). Our results are largely consistent with previous research examining the feasibility to work from home across occupations, economic activities and population groups (Dingel and Neiman 2020; Delaporte and Peña 2020; Gottlieb et al. 2021; Hatayama et al. 2020).

### The Lockdown Working Ability (LWA) index under perfect compliance

The stay-at-home orders do not apply to all economic activities. Certain activities have remained open, either because they are considered as essential or because some LAC countries have implemented a partial lockdown. On the opposite, other activities have ceased completely to operate. Lastly, some activities that are not explicitly stated as essential or closed have been affected by mobility restrictions. This needs to be taken into account when estimating the share of individuals able to remain active under the lockdown. Therefore, following Palomino et al. (2020) and Duman (2020), we construct the LWA index under perfect compliance which can be expressed as follows:

1 $${LWA}_{i}=\left\{\begin{array}{cc}1& if{\:}\mathrm{ }{a}_{i}=O\\ {T}_{i}& if{\:}\mathrm{ }{a}_{i}\ne O,\mathrm{ }C\\ 0& if{\:}\mathrm{ }{a}_{i}=C\end{array}\right.$$

where $O$ refers to open economic activities and $C$ to closed economic activities. ${T}_{i}$ refers to the teleworkability index of individual $i$. In other words, when a certain economic activity is open, we assume that the workers are not affected by the lockdown regardless of their capacity to work from home. On the opposite, when a certain economic activity is closed, we assume that working is not possible, regardless of the fact that the job can be performed at home. The feasibility to work from home matters only for the remaining economic activities.

Our LWA measure might have some limitations which should be underlined. First, we assume that all individuals working in sectors that are open are able to remain active. This might not be the case if the jobs in sectors that are open are affected by a drop in demand or by distancing measures at work (some workers could have been fired or furloughed).^14^ Besides, we assume that the workers in these sectors retain their pre-lockdown level of hours worked per week. We also acknowledge the possibility that some workers in the sectors that are open might perform their job from home. This, however, does not affect our conclusions since, no matter where they decide to work, the number of hours worked should remain the same, as their labour income.

Second, for individuals who work in sectors that are closed, we assume that they can no longer work and therefore do not receive their salary. This might not be the case for all individuals. Some workers might have continued to receive their salary, regardless of the fact that their occupation is affected by legal workplace closures, either because of the rigidity of their contracts or because they are able to perform certain tasks from home. In this case, they would have received the totality or part of their salary.^15^ Finally, in the case of the remaining sectors that are affected by mobility restrictions, we assume that all the workers that are able to work from home do so. Yet, some workers might have been fired or furloughed irrespective of their ability to work from home.

To proceed with the estimation of the LWA index, we need to classify the economic activities into three categories: (i) the activities that are explicitly stated as closed, (ii) the activities that are explicitly stated as open and (iii) the remaining activities affected by mobility restrictions. We gather detailed information from national laws, decrees and press releases for each country in our sample. The decisions to close down or leave open specific economic activities have been taken at the sectoral level. Therefore, we conduct this classification at the sectoral level. The only available variable for sectors that has been harmonised by the IADB in the household surveys is at the 1-digit level. This gives us nine different sectors.^16^ Having such a general definition of the economic activities does not allow us to identify precisely which sectors were closed or open. Therefore, we use the non-harmonised version of the sectoral classification available in each survey which is more detailed and employ a crosswalk between the national classifications (often at the 4-digit level) and the harmonised classification ISIC revision 4. For some countries, we use a crosswalk from the national classification to ISIC Rev 3.1 and then to ISIC Rev 4.^17^ By following this procedure, we obtain a more detailed and harmonised definition of economic activities (at the 2-digit or division level).

We now proceed with the classification of the sectors into open and closed activities. Since countries in the LAC region have implemented different lockdown policies, it is important to identify in each country which sectors are open and which are closed, as well as the duration of the first phase of the lockdown.^18^ The estimated start date of the lockdown is the date at which the country entered into a lockdown while the estimated end date of the lockdown is the date at which considerable reopening of industry and/or services took place under certain conditions. Some countries have adopted a regional approach where the lockdown measures differ across regions. This is the case of Brazil for which we identify the classification of the sectors as well as the duration of the lockdown at the state level. For other countries such as Argentina and Chile, the duration of the lockdown differs across regions but the classification of the sectors remains the same.

**Fig. 2:**
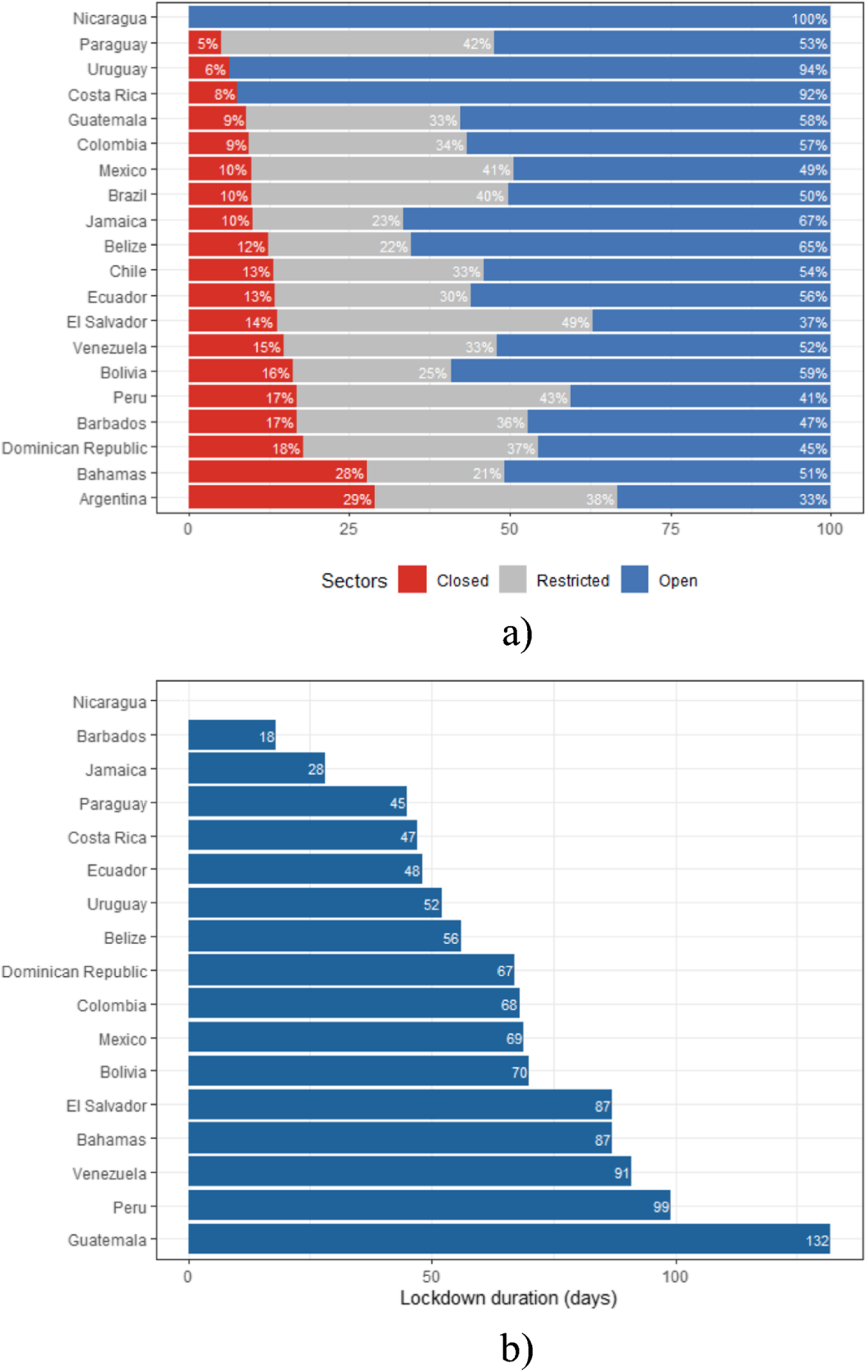
Lockdown intensity and duration, by country. **a** Share of workers in closed, restricted and open sectors, by country. **b** Lockdown duration, by country. Source: Harmonised Household Surveys of Latin America and the Caribbean, and National Laws, Decrees and Press Releases, authors’ own calculations. Notes: In a, we report for Brazil the classification that is in place in Brazil’s capital. Argentina, Brazil and Chile are not included in b since they did not adopt a national approach

Figure 2 presents the lockdown intensity and duration by country. In particular, Fig. 2a reports for each country the proportion of workers in (i) sectors that are closed, (ii) sectors affected by mobility restrictions and (iii) sectors that are open.^19^ Figure 2b reports the estimated lockdown duration (in days) for all the countries that have adopted a national approach.^20^

We find strong differences across countries in the type of lockdown that was implemented. Nicaragua is the only country in our sample that did not implement a lockdown. Therefore, all the workers in Nicaragua are potentially able to remain active. Paraguay and Uruguay have the lowest proportions of workers in sectors that are closed. Yet, Paraguay has imposed a stricter lockdown than Uruguay. As a result, almost all the workers in Uruguay are potentially able to remain active under the lockdown, whereas in Paraguay, it depends additionally on the capacity of individuals to work from home. On the other extreme of the spectrum, the Bahamas and Argentina have the highest proportions of individuals that are working in closed sectors (28% and 29% respectively). Countries such as the Dominican Republic, Barbados or Peru also have a high proportion of workers that are unable to work (around 17–18%). Lastly, the highest proportion of workers in sectors that are affected by mobility restrictions is found in El Salvador with 49% of the workers required to work from home. Therefore, the ability to remain active for these workers rests essentially on their capacity to perform their job from home. There are also strong differences across countries in terms of the duration of the first phase of the lockdown. In this respect, Guatemala has implemented the longest lockdown at the national level (132 days).

**Fig. 3:**
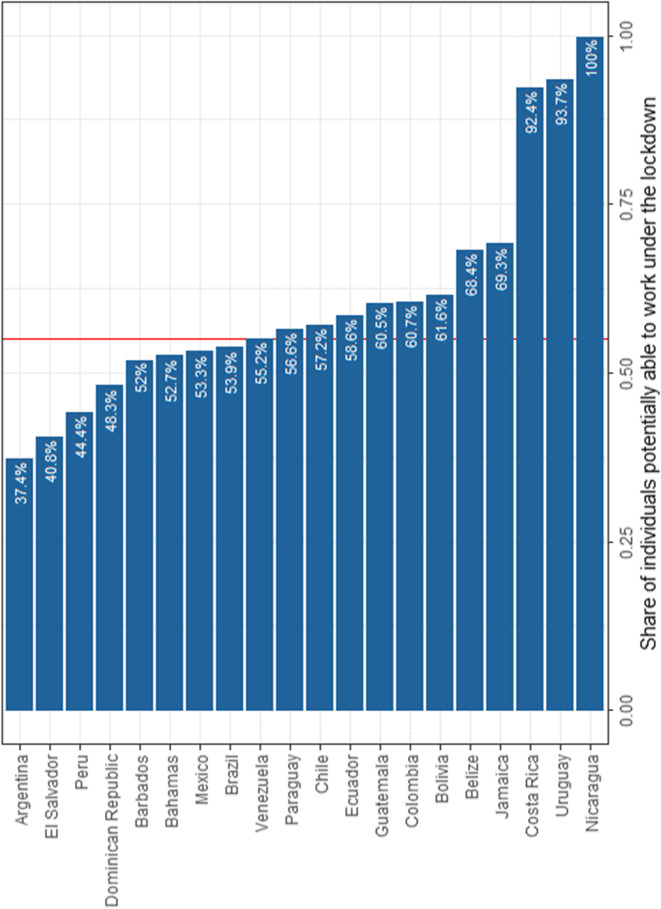
Share of individuals able to work under the lockdown under perfect compliance, by country. Source: Harmonised Household Surveys of Latin America and the Caribbean, authors’ own calculations. Notes: The average share of individuals able to work from home (represented by the vertical red line) is 55% for the entire LAC region. The LAC share was calculated as a weighted average of the population in all countries, excluding Argentina and the Bahamas, which are not representative at the national level, and Nicaragua to be consistent with the scenario under imperfect compliance

Based on the sectoral classifications and on individuals’ teleworking capacity, we can now construct the LWA index. Figure 3 reports the share of individuals potentially able to remain active under the first phase of the lockdown in each country. These proportions differ from the ones reported in Fig. 2a since the individuals who are able to remain active can be among (i) the individuals who work in sectors that are open and (ii) the individuals who are able to work from home among those who work in sectors that are affected by mobility restrictions. The results show that, on average, 1 worker out of 2 is potentially able to work under the lockdown in the entire LAC region. This proportion varies from 37% in Argentina to 100% in Nicaragua.

We examine further how this share varies within countries across occupations, economic activities and specific population groups. The share of individuals able to work under the lockdown differs across occupations (Appendix, Table 7). The highest share of workers potentially able to remain active is found among skilled agricultural, forestry and fishery workers (97% for the full sample). A high share is found as well among professionals (82% for the full sample), clerical support workers (72% for the full sample), technicians and associate professionals (63% for the full sample) and managers (56% for the full sample). On the opposite, individuals who work as craft and related trades workers as well as plant and machine operators and assemblers are less likely to be able to work under the lockdown (23% and 42% respectively for the full sample). Unsurprisingly, there are important differences across countries since it depends on the strictness of the lockdown. For instance, only 50% of the workers in agriculture are able to remain active in the Bahamas, compared to 100% of their peers in Nicaragua.

The proportion of individuals potentially able to work during the lockdown also varies across economic activities (Appendix, Table 8). The highest share is found in agriculture as well as in the electricity, gas and water sector (100% for the full sample). This result does not differ much across countries. A significant share of individuals are able to work as well in mining and quarrying (88% for the full sample), in financial insurance and real estate (65% for the full sample) and in the transport and storage sector (65% for the full sample). These shares differ however substantially depending on the country that is examined. The lowest share of individuals able to work during the lockdown is found in the construction sector (11% for the full sample).

Lastly, we investigate how the capacity to work under the lockdown differs across individuals (Appendix, Table 9). A larger proportion of men are able to work during the lockdown compared to women (56% compared to 53% for the full sample). Besides, a higher proportion of highly educated individuals are able to remain active compared to individuals with lower educational attainment (57% compared to 52% for the full sample). Individuals living in rural areas are more likely to be able to remain active compared to workers in urban areas (72% compared to 51% for the full sample). A higher proportion of formal workers are able to remain active as well compared to informal workers (60% compared to 50% for the full sample). Lastly, a larger share of individuals in the top of the labour income distribution are able to remain active under the lockdown compared to those in the bottom part (63% compared to 55% of the individuals in the bottom quintile for the full sample).

### The Lockdown Working Ability (LWA) index under imperfect compliance

We have assumed so far that the lockdown policies have been fully enforced and respected in all LAC countries. In other words, we have assumed that de facto and de jure lockdowns are the same; thus, there is perfect compliance. However, a number of recent studies have shown that de facto lockdowns are very different from de jure lockdowns, especially in developing countries (Maloney and Taskin 2020; Galasso et al 2020). Indeed, government capabilities to enforce are weaker, and resistance is often higher since the trade-off with livelihood is harsher in developing countries. Yeyati and Sartorio (2020a) show that people’s capability and willingness to comply with restrictive policies is lower in countries with lower incomes and higher levels of labour precariousness. It is also lower in countries with stricter and longer quarantines. Besides, compliance is related to the pre-crisis level of trust in policy makers (Bargain and Aminjonov 2020) and to pre-crisis social responsible behaviour (Müller and Rau 2021), which differ across countries. Therefore, assuming perfect compliance is likely to lead to biased estimates. To address this concern, the degree of non-compliance should be taken into account when examining the feasibility to remain active under a lockdown.

In order to compare the stringency of de jure lockdown policies with de facto compliance over time, we use the Oxford Stringency Index (OSI) compiled by the University of Oxford as well as Google workplace mobility data.^21^ More specifically, we rely on the OSI since it provides a continuous measure of the strictness of the lockdown policy implemented in all LAC countries. This allows us to examine the evolution in the level of stringency of de jure lockdowns over time. With respect to mobility data, we focus on workplace mobility since it is the type of mobility that is arguably the most closely related to the economic costs of the pandemic (Yeyati and Sartorio 2020a). The Google Mobility Index (GMI) estimates the variation of mobility relative to a baseline date previous to the pandemic. By comparing the two measures over time, this gives us an idea of the evolution of de facto compliance.

**Fig. 4:**
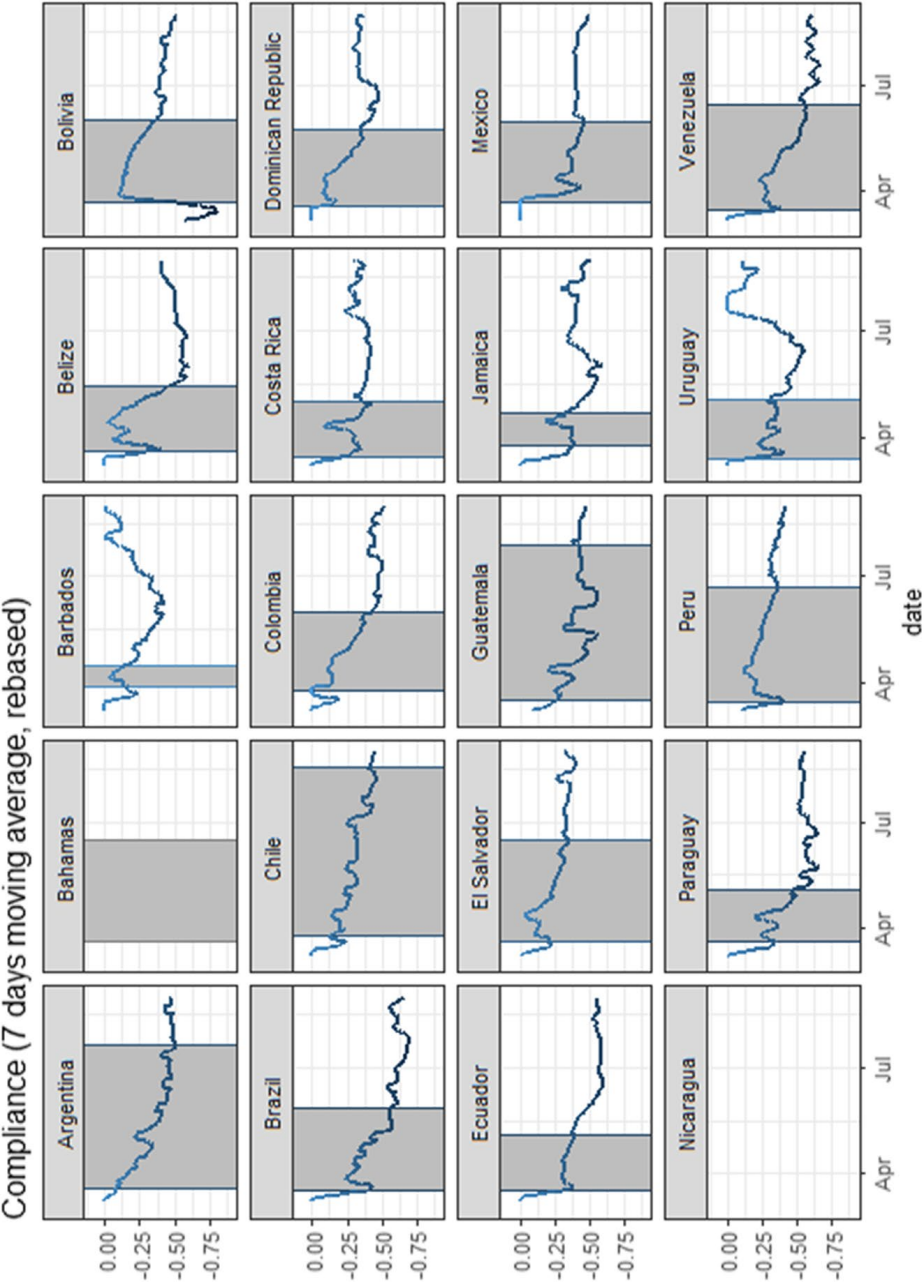
Evolution of lockdown compliance, by country. Source: Oxford Stringency Index and Google Workplace Mobility Data, authors’ own calculations. Notes: Compliance is computed by subtracting the normalised Oxford Stringency Index (OSI) from the normalized Google Mobility Index (GMI). A negative number indicates non-compliance. Both indices were normalized to 0 on March 3, 2020. Since compliance is conditional on having a lockdown, changes in lockdown compliance cannot be observed for Nicaragua. Furthermore, the OSI has not been computed for the Bahamas. Therefore, compliance cannot be observed. The grey shaded area represents the first phase of the lockdown in all countries. For countries that have adopted a regional approach, we use the lockdown duration of the capitals

It should be noted that the GMI is reported at the regional level for each country. However, the OSI is reported at the national level for all LAC countries (except for Brazil, where the index differs across states). Therefore, in order to compute the degree of compliance at the regional level in each country, we have to assume a common stringency index across regions. Following Yeyati and Sartorio (2020a), we normalize both the OSI and the GMI to 0 on March 3, 2020. This allows us to compare the evolution of de jure and de facto lockdowns both at the regional level within countries and at the national level across countries. Then, we can subtract the normalised OSI from the normalised GMI in order to estimate the degree and evolution of compliance across regions and countries. Figure 4 reports the degree and evolution of compliance over time in each country. The grey shaded area represents the first phase of the lockdown in all countries.^22^

The level of compliance generally varies over time in most of the countries. An increase in non-compliance can be observed when the level of stringency of the lockdown policy was higher than the drop in work mobility. Overall, lockdown compliance has steadily decreased in many countries over the period of the pandemic. Towards the end of the first phase of the lockdown, most of the countries experience a decrease in compliance. The highest drop in compliance is registered in Brazil and Venezuela (− 58% and − 57% respectively). Furthermore, the drop in compliance seems to be faster in some countries than others as time passes by. As previously mentioned, there are many potential reasons behind these differences in the level of compliance across countries.^23^ We document that countries characterised by lower levels of development, weak institutionality and higher levels of labour precariousness, in particular with a larger share of workers in informality, are more likely to have lower levels of compliance, a phenomenon that was also documented by Yeyati and Sartorio (2020a). Second, countries that have implemented longer and stricter quarantines are more likely to have a lower level of compliance as time goes by. In addition, countries with limited coverage of social assistance programmes during the pandemic might have experienced faster drops in compliance compared to countries with higher coverage. Another potential explanation is the way political leaders have framed the pandemic in their public speeches (Ajzenman et al. 2020).

Therefore, assuming perfect compliance is likely to lead to a biased estimation of the potential share of individuals who are able to remain active. In order to allow for some degree of non-compliance, we modify the expression of the Lockdown Working Ability measure as follows:

2 $${LWA}_i^{NC}=\left\{\begin{array}{c}1\;if\;a_i=O\\T_i+\left(1-T_i\right){NC}_j\;if\;a_i\neq O,C\\0+{NC}_{j\;}if\;a_i=C\end{array}\right.$$

where $O$ refers to open economic activities and $C$ to closed economic activities. ${T}_{i}$ refers to the teleworkability index of individual $i$ and ${NC}_{j}$ to non-compliance in country $j$. In other words, when a certain economic activity is open, we assume that the workers are not affected by the lockdown regardless of their capacity to work from home. On the opposite, when a certain economic activity is closed, we assume that although individuals are not supposed to work, a proportion of workers remain active due to non-compliance. Lastly, for the remaining activities, individuals who can work from home remain active. In addition, among those who cannot work from home, we assume that a share of individuals remain active due to non-compliance.

To include non-compliance in our LWA estimation, we use the lockdown compliance index presented in Fig. 4. More specifically, since the level of compliance varies over time and we calculate our LWA at one point in time, we proxy non-compliance as the average over the period of the first phase of the lockdown in each region within countries. At the national level, our non-compliance measure indicates that non-compliance varies on average from 10% in Barbados to 45% in Guatemala during their respective lockdowns. We also implicitly assume that the value of the index can be a proxy for the percentage of people required to stay at home that are not complying with the lockdown measures.^24^ In addition, we assume that this level of non-compliance is the same across sectors. In other words, workers within closed sectors and workers in sectors that have been affected by mobility restrictions are both likely not to comply.^25^ We consider this procedure to be consistent with our framework and intuitive enough (as we do not make further assumptions by imposing restrictions on the nature of non-compliance) to unveil the role of non-compliance in the ability to remain active under lockdowns and to understand the links between non-compliance and socio-economic background. However, we acknowledge that this exercise has some limitations apart from the ones already mentioned. One of them is that we might overestimate the potential share of individuals able to remain active under the lockdown. Therefore, the shares should be interpreted as upper bounds of the true proportion of workers that remain active.

**Fig. 5:**
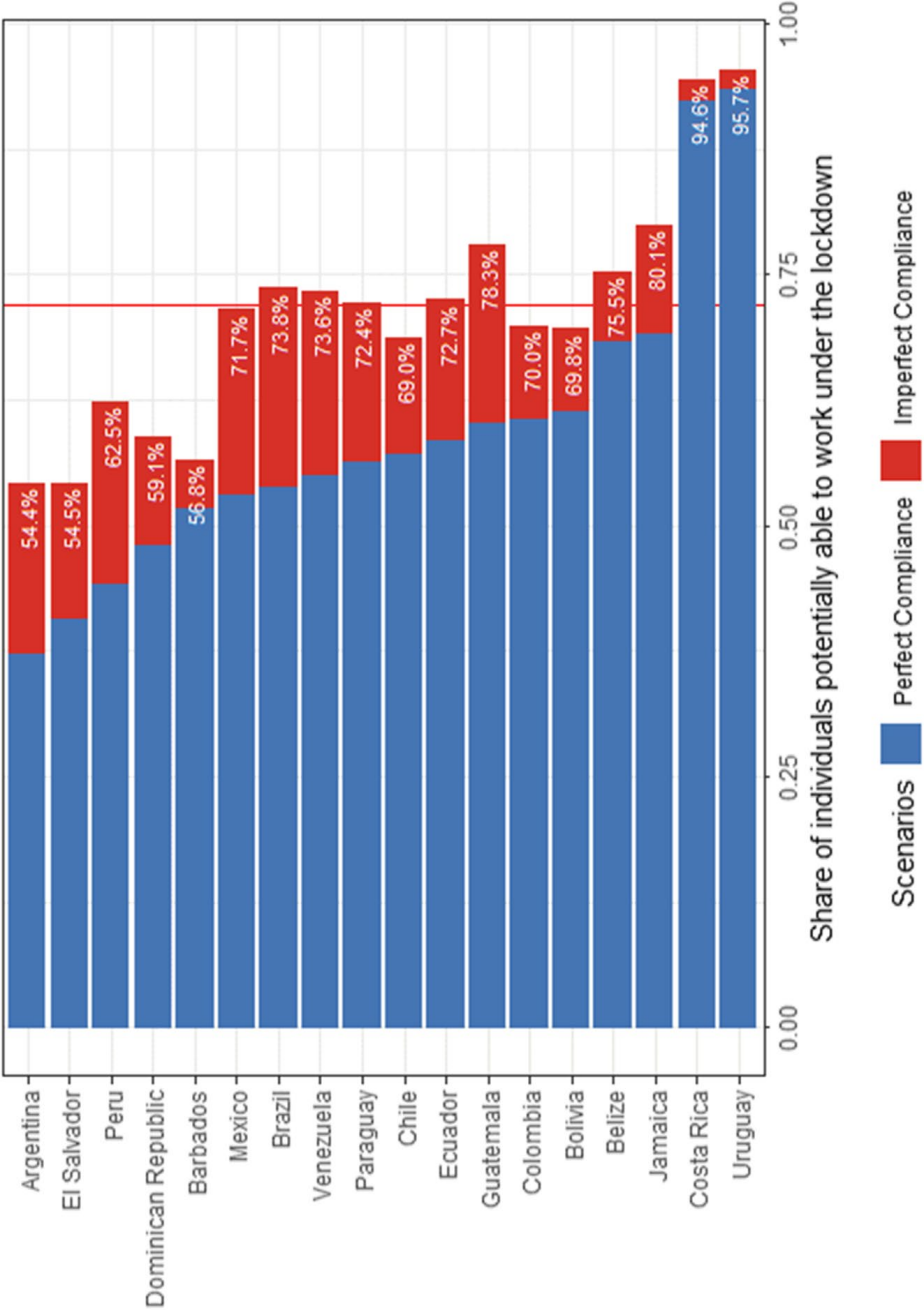
Share of individuals able to work under the lockdown under imperfect compliance, by country. Source: Harmonised Household Surveys of Latin America and the Caribbean and Oxford Stringency Index and Google Workplace Mobility Data, authors’ own calculations. Notes: Two countries are not included: the Bahamas and Nicaragua. Since compliance is conditional on having a lockdown, lockdown compliance cannot be observed for Nicaragua. Furthermore, the OSI has not been computed for the Bahamas. The average share of individuals able to work under the lockdown (represented by the vertical red line) is 72% for the entire LAC region. The LAC share was calculated as a weighted average of the population in all countries, excluding Argentina and the Bahamas, which are not representative at the national level, and Nicaragua to be consistent with the scenario under imperfect compliance

We now compute the LWA measure under de facto compliance (Fig. 5). The proportions represented in blue are the same shares documented previously under perfect compliance while the added proportions in red are for each country the additional share of individuals that are potentially able to remain active under the lockdown due to non-compliance.

We find large differences across countries since LAC countries exhibit very different levels of compliance; yet, when we take non-compliance into account, the potential ability to work during the lockdowns increases in all countries. Non-compliance is the lowest in countries such as Costa Rica and Uruguay. Among other reasons, this is likely due to the type of lockdown policy that was implemented. Costa Rica and Uruguay did not implement a strict lockdown compared to other LAC countries, thus decreasing the need and urge for individuals not to comply. In the opposite, countries such as Brazil and Venezuela have higher levels of non-compliance. As a result, the additional share of individuals potentially able to remain active due to a scenario of imperfect compliance is higher in these countries. Overall, by taking into account the possibility that individuals do not comply with the social distancing rules, we find that the proportion of individuals potentially able to remain active varies from 54% in Argentina to 96% in Uruguay.^26^ At the level of the LAC region, the average share of individuals potentially able to work is 72%.

We examine further how the incremental shares differ within countries across occupations, economic activities and specific population groups. This exercise also allows us to examine the characteristics of potential non-compliers. The increase in the share of individuals potentially able to work under the lockdown due to de facto compliance differs across occupations (Appendix, Table 10). The highest increase in the proportion of workers able to remain active due to imperfect compliance is found among craft and related trades workers (28 pp increase for the full sample). A significant increase is found as well among service and sales workers (22 pp for the full sample), plant and machine operators and assemblers (21 pp for the full sample) and workers in elementary occupations (21 pp for the full sample). On the opposite, the share of active workers among professionals and technicians and associate professionals is increasing to a lower extent due to non-compliance. Overall, we document that potential non-compliers are concentrated, on average, in low-skilled occupations.

The increase in the potential to work during the lockdown also varies across economic activities (Appendix, Table 11). The highest increase in the proportion of workers able to remain active due to imperfect compliance is found in the construction sector (31 pp increase for the full sample) as well as in manufacturing industries (24 pp increase for the full sample). There is no significant change in the proportion of individuals that are able to remain active in agriculture and in the electricity, gas and water sector.

Lastly, we investigate how the incremental shares differ across individuals (Appendix, Table 12). As a result of non-compliance, a similar increase is observed among men and women on average (around 17–18 pp increase for the full sample). As expected, there is a higher level of non-compliance among individuals that have a lower level of education and who live in urban areas. There is also a higher increase in the share of informal workers compared to formal workers (18 pp increase compared to 16 pp increase). Lastly, a higher increase is observed among individuals in the bottom part of the total labour income distribution compared to individuals in the top part of the total labour income distribution (17 pp increase compared to 14 pp increase). Overall, our results are consistent with those one would expect: the potential non-compliers are among the most vulnerable individuals and are the ones that have been the most affected by the social distancing rules.

## Poverty and inequality changes due to COVID-19

The asymmetry of the shock implies that the economic implications of social distancing could be significant in terms of labour income inequality and poverty rates. In this section, we partly follow Palomino et al. (2020). In particular, we calculate similar inequality measures. However, we do not simulate changes under different scenarios of lockdown intensity and duration. We conduct an ex-post assessment of the potential effects of the lockdown policies applied in each LAC country on poverty and labour income inequality. Furthermore, we compare the formal (de jure) lockdown policies with de facto compliance. The inclusion of imperfect compliance in our analysis allows to uncover the potential role of non-compliance as a mechanism to smooth labour income losses related to lockdowns. Before presenting our analysis, it should be noted that we focus on the potential impact of the first phase of the lockdown and do not consider the potential effect of the subsequent phases that have for objective to organise the reopening of the economies, neither the possibility of a second lockdown. In addition, our analysis is framed in a partial equilibrium setting since we do not take into account other effects that might have impacted the labour income distribution.

### Potential labour income losses and inequality measures

To examine the potential impact of enforced social distancing on poverty and labour income inequality, the first step is to calculate the potential labour income loss due to the lockdown for all individuals. The labour income loss is calculated as follows:

3 $${wl}_{it}= {w}_{it-1} . {D}_{j} (1- {LWA}_{i})$$

where ${wl}_{it}$ is the labour income loss of individual $i$ in period $t$. ${w}_{it-1}$ is the annual labour income^27^ of individual $i$ in period $t-1$ (before the lockdown) and ${D}_{j}$ represents the duration of the lockdown in annual terms in country $j$, i.e. ${D}_{j}= \frac{30}{365}$ for 30 days, ${D}_{j}= \frac{60}{365}$ for 60 days, etc. The duration of the lockdown differs across countries and, for some countries, across regions.^28^ Lastly, ${LWA}_{i}$ represents the capacity for individual $i$ to remain active and to receive his salary during the period of the lockdown.^29^ We estimate the labour income losses subsequently under two scenarios: (i) under perfect compliance with ${LWA}_{i}$ and (ii) under imperfect compliance with ${LWA}_{i}^{NC}$. To provide an example, for individuals that work in open sectors, the LWA index is assumed to be 1. Therefore, the expression for the labour income losses is equal to 0. This means that the individuals that are able to remain active under the lockdown do not experience labour income losses. Thus, the labour income loss experienced by workers under a lockdown is the proportion of annual labour income they lose due to their inability to work during the lockdown period. The estimated labour income losses allow us to evaluate the potential changes in poverty and labour income inequality under the first phase of the lockdown in each LAC country.

An important point to note here is that, since we focus our attention on labour income, we do not capture the effects of government transfers and subsidies put in place to help households and individuals. Such effects would be captured at the household income level. In addition, we are not capturing the support of some governments to pay a share of the payroll of some formal employees. However, knowing whether and to which extent aid programmes (for employment or income protection) were implemented during the pandemic in the LAC region can be informative for our study. Therefore, we provide a summary of the programmes which were explicitly targeting informal workers or aiming at having an effect on the labour income of formal workers in the Appendix, Table 13. Busso and Messina (2020) and Busso et al. (2020) discuss the generosity of the emergency transfers in 10 LAC countries and find a good potential coverage among the poorest households, reaching more than 75% of the poorest tercile in the population in most countries. However, coverage is lower in the second tercile.^30^ Therefore, these emergency measures should be kept in mind since they are likely to attenuate some of the distributive effects of the lockdown policies.

To examine the changes in poverty and inequality, we calculate a series of measures which we define, before presenting the results. First, we calculate the loss rate in the labour income of every worker caused by the lockdown, i.e. ${l}_{it}= \frac{{w}_{it}- {w}_{it-1}}{{w}_{it-1}}= \frac{- {wl}_{it}}{{w}_{it-1}}$ with ${w}_{it}= {w}_{it-1}- {wl}_{it}$. We order individuals by their pre-lockdown labour income and group them into percentiles, obtaining the mean loss rate at each percentile. This gives us the Lockdown Incidence Curve (LIC), which allows us to examine which part of the labour income distribution suffers the largest relative loss. In other words, it provides a simple illustration of the changes between the pre-lockdown period and the period at the end of the first phase of the lockdown for each percentile.^31^ We estimate two LIC curves for each country: the first one under perfect compliance and the second under imperfect compliance. This allows us to compare the distributional effects of the formal de jure lockdowns with de facto compliance. We also examine how the mean loss labour income rate varies across occupations, economic activities and population groups under the two scenarios.

Then, we calculate the Foster-Greer-Thorbecke indices which are a family of poverty metrics. These indices are derived by substituting different values of the parameter $\propto$ into the following equation:

4 $${FGT}_{\propto }= \frac{1}{N} \sum _{i=1}^{H}{\left(\frac{z - {w}_{i}}{z}\right)}^{\propto }$$

where $$z$$ is the poverty threshold; N is the number of people in the economy; H is the number of poor (those with labour incomes at or below $$z$$); $${w}_{i}$$ is the labour income of each individual $$i$$. If $$\propto$$ is low, the FGT metric weights all the individuals with incomes below $$z$$ roughly the same. The higher the value of $$\propto$$, the greater the weight placed on the poorest individuals. We calculate $${FGT}_{0}$$, $${FGT}_{1}$$ and $${FGT}_{2}$$. $${FGT}_{0}$$ is the headcount ratio. It is the fraction of workers that live below the international poverty line of $5 (PPP) per person per day.^32^ With $$\propto =1$$, $${FGT}_{1}$$ is the poverty gap index. Lastly, $${FGT}_{2}$$ measures the intensity/severity of poverty. We compute these measures for the pre-lockdown period as well as for the period at the end of the first phase of the lockdown.^33^ In addition, we calculate the absolute changes denoted as follows: $${\Delta }^{A}{FGT}_{0}$$, $${\Delta }^{A}{FGT}_{1}$$ and $${\Delta }^{A}{FGT}_{2}$$. Lastly, we calculate the measures subsequently under perfect and imperfect compliance.

With respect to changes in labour income inequality, we calculate the Gini coefficient (G) and the Mean Logarithmic Deviation (MLD) index. The Gini coefficient can be expressed as follows:

5 $$G\left(w\right)= \frac{1}{2{n}^{2}\mu } \sum _{i=1}^{n}\sum _{k=1}^{n}|{w}_{i}- {w}_{k}|$$

where $w$ represents the labour income distribution, ${w}_{i}$ is the labour income of individual $i$ and $\mu$ is the mean labour income of the economy. The absolute changes in labour income inequality are measured as the difference between the pre-lockdown labour income distribution and the labour income distribution at the end of the first phase of the lockdown: ${\Delta }^{A}G=G\left({w}_{t}\right)- G\left({w}_{t-1}\right)$, while the relative changes in labour income inequality are measured as percentages of pre-lockdown inequality, i.e. ${\Delta }^{R}G= \frac{G\left({w}_{t}\right)- G\left({w}_{t-1}\right)}{G\left({w}_{t-1}\right)} \times 100.$ We also estimate these changes subsequently under perfect and imperfect compliance. Second, we use the MLD index which can be expressed as follows:

6 $$MLD\left(w\right)= \frac{1}{n} \sum _{i=1}^{n}\mathrm{ln}(\frac{\mu }{{w}_{i}})$$

We compute the absolute and relative changes in labour income inequality measured by the MLD index which will be denoted by ${\Delta }^{A} MLD=MLD\left({w}_{t}\right)-MLD({w}_{t-1})$ and ${\Delta }^{R} MLD= \frac{MLD\left({w}_{t}\right)-MLD({w}_{t-1})\mathrm{ }}{MLD({w}_{t-1})} \times 100,$ respectively. Similarly, we estimate these changes subsequently under perfect and imperfect compliance. Lastly, the MLD index can be decomposed into a between-group and a within-group component. While the between-group component is the level of labour income inequality that would arise if each worker in a country enjoys the mean labour income of the country, the within-group component is the weighted sum of labour income inequalities within different countries. We conduct the decomposition in order to estimate the relative contribution in overall inequality.

### *Impact of de jure and *de facto* lockdown policies on poverty and inequality*

**Fig. 6:**
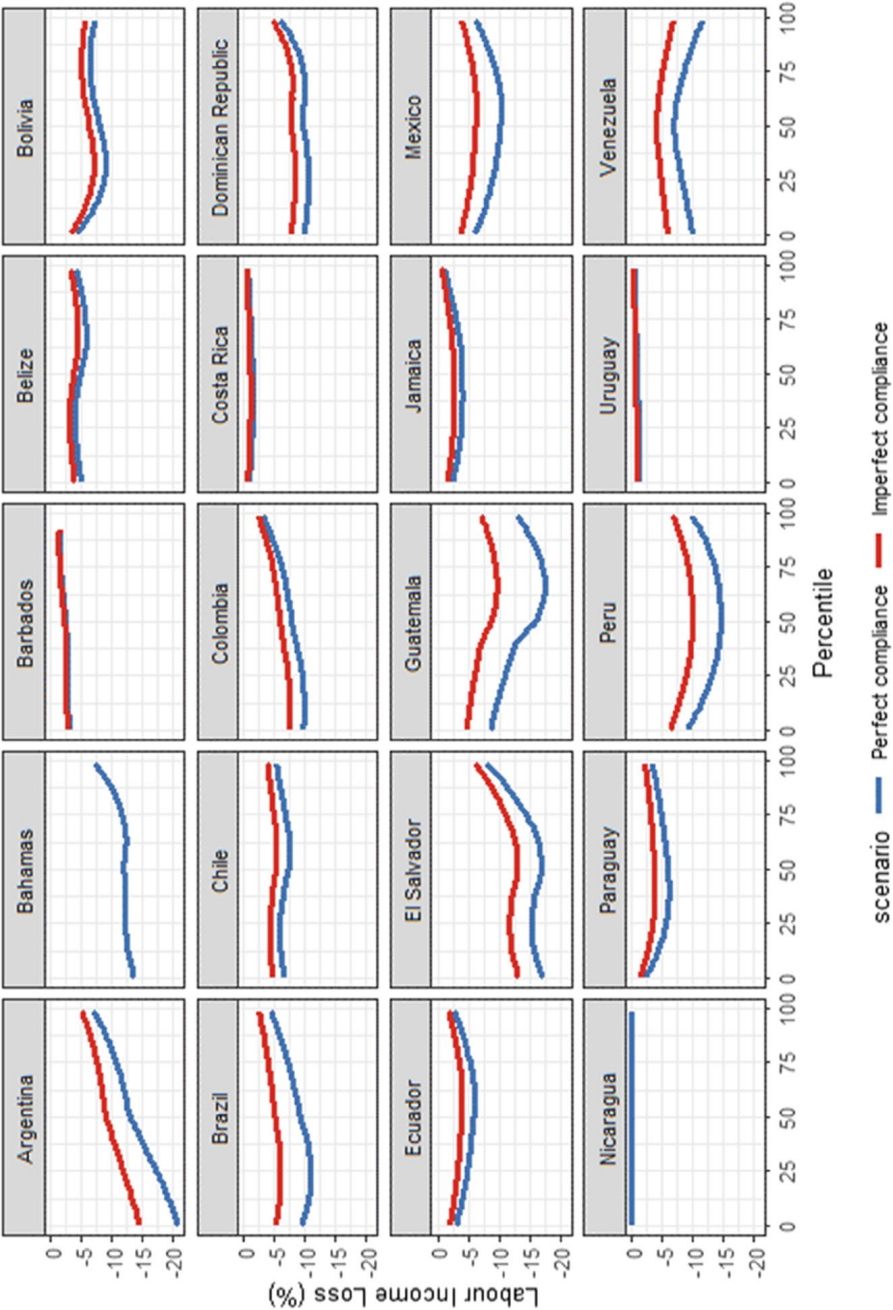
Lockdown incidence curves under perfect and imperfect compliance, by country. Source: Harmonised Household Surveys of Latin America and the Caribbean and Oxford Stringency Index and Google Workplace Mobility Data, authors’ own calculations. Notes: The blue curves represent the relative change in the annual labour income distribution assuming perfect compliance, while the red curves represent the relative changes in the annual labour income distribution when allowing for some degree of non-compliance. The LIC curves allow examining which part of the labour income distribution suffers the largest relative labour income losses. A smoother has been applied to the curves

We first examine the LIC curves for each LAC country of our sample. Figure 6 provides the LIC curves under perfect compliance (blue curves) and the LIC curves under imperfect compliance (red curves). If each percentile of the earnings distribution — i.e. each 1% of the population earning a labour income ordered from the lowest to the highest group — was experiencing equal labour income losses, then the LIC curve would be represented by a straight line. Furthermore, if the labour income loss is decreasing across the labour income distribution, then inequality rises with the lockdown for all inequality measures satisfying the Pigou–Dalton transfer principle.

When assuming perfect compliance, the picture differs across countries. Indeed, there is a lot of heterogeneity across countries in terms of the levels and the slopes of the incidence curves. First, not all parts of the labour income distribution are affected similarly in all countries. For instance, in Argentina, the Bahamas, Barbados, Brazil, Colombia, Dominican Republic and El Salvador, the bottom percentiles are the most affected. By contrast, in Belize, Chile, Costa Rica, Ecuador, Jamaica, Paraguay and Uruguay, all parts of the labour income distribution suffer relatively similar losses. Furthermore, when the bottom percentiles are affected, they are not affected in similar proportions in all countries. They are more disproportionately impacted in Argentina and in El Salvador compared to those in the Bahamas, Barbados, Brazil, Colombia and Dominican Republic. In Mexico, Paraguay and Peru, there is an increase in the labour income polarization since the most affected are individuals in the middle part of the labour income distribution.

When comparing de jure with de facto lockdowns, labour income losses are higher when assuming perfect compliance. However, the differences depend on the level of non-compliance. Not all countries have a significant degree of non-compliance; and therefore, for these countries, we do not observe significant differences. This is the case for Barbados, Belize, Bolivia, Costa Rica, Ecuador, Jamaica, Paraguay and Uruguay. By contrast, the differences between de jure and de facto lockdowns are more striking in the case of Argentina, Brazil, El Salvador, Guatemala, Mexico, Peru and Venezuela. This is potentially due to differences in the informal employment rate — it is above 70% in El Salvador, Guatemala and Peru. In all these countries, we can see that non-compliance attenuates the labour income losses of those that are the most affected.

Lastly, as mentioned previously, we do not include social assistance programmes that were implemented during the pandemic in the LAC region. However, since we know that the emergency transfers target primarily the poorest individuals, we can expect the labour income loss rate to be reduced in the lower part of the total labour income distribution.

To identify the potentially most affected individuals in the population, we estimate the mean loss labour income rate under perfect compliance across occupations, economic activities and population groups. Among all occupations (Appendix, Table 14), craft and related trades workers suffer the largest losses. In the LAC region on average, their potential labour income loss amounts to 15% of their pre-lockdown annual labour income. Across the different economic activities (Appendix, Table 15), workers in the construction sector, in manufacturing industries and in the wholesale and retail trade sector experience important labour income losses as well. We also examine how the relative labour income losses vary across different population groups (Appendix, Table 16). The potential labour income losses do not differ significantly by gender as well as by level of education. However, informal workers have higher potential labour income losses than formal workers (10% compared to 8% for the full sample). Besides, workers in urban areas experience higher potential labour income losses (10% compared to 6% in rural areas for the full sample).

When allowing for some degree of non-compliance, the mean loss labour income rates are reduced. We examine the reduction in the mean loss labour income rate due to imperfect compliance across occupations, economic activities and population groups. First, across all occupations (Appendix, Table 17), craft and related trades workers would experience a lower drop of their pre-lockdown annual labour income due to imperfect compliance. This is due to the fact that these workers are among those with higher probability of non-compliance. Across economic activities (Appendix, Table 18), workers in the construction sector also experience a lower drop in their labour income on average. Lastly, when examining differences across individuals (Appendix, Table 19), a larger reduction in the labour income losses of informal workers is observed compared to formal workers. This directly comes from the fact that informal workers are more likely not to comply with social distancing rules than formal workers.^34^

Our results for the analysis on poverty and labour income inequality are reported in Table 1.^35^ We find an increase in the proportion of workers with a labour income below the international poverty line of $5 (PPP) in almost all the LAC countries. While the average increase in the headcount poverty index is of 1.6 pp for the LAC region when assuming perfect compliance, this increase is reduced to 0.8 pp when considering imperfect compliance. Therefore, the changes are significantly reduced under imperfect compliance. This is due to the fact that the potential non-compliers are among the most vulnerable individuals. However, we still observe an increase in the proportion of workers considered as poor in most of the countries. At the national level, the highest increase in the headcount poverty index when assuming perfect compliance is observed in Guatemala (6 pp increase). However, under imperfect compliance, the highest increase is observed in El Salvador (2.5 pp increase).

**Table 1:** Poverty and labour income inequality changes, by country

Countries	Changes under perfect compliance							Changes under imperfect compliance						
	${\Delta }^{A }{FGT}_{0}$	${\Delta }^{A }{FGT}_{1}$	${\Delta }^{A }{FGT}_{2}$	${\Delta }^{A }G$	${\Delta }^{R}G (\%)$	${\Delta }^{A }MLD$	${\Delta }^{R}MLD (\%)$	${\Delta }^{A }{FGT}_{0}$	${\Delta }^{A }{FGT}_{1}$	${\Delta }^{A }{FGT}_{2}$	${\Delta }^{A }G$	${\Delta }^{R}G (\%)$	${\Delta }^{A }MLD$	${\Delta }^{R}MLD (\%)$
All	0.016	0.007	0.005	0.012	2.5	0.021	4.3	0.008	0.004	0.003	0.006	1.3	0.010	2.1
Argentina ^a^	0.036	0.013	0.008	0.029	7.0	0.047	13.7	0.025	0.009	0.005	0.019	4.5	0.030	8.8
Bahamas ^a^	0	0.003	0.002	− 0.002	− 0.4	− 0.007	− 1.3							
Barbados	0	0	0	0.004	1.3	0.003	1.9	0	0	0	0.003	1.2	0.003	1.7
Belize	0.003	0.001	0	0.001	0.2	0.003	0.3	0.003	0.001	0	0.001	0.1	0.002	0.2
Bolivia	0.007	0.004	0.002	0.006	1.5	0.005	1.4	0.006	0.003	0.002	0.004	1.0	0.003	0.8
Brazil	0.013	0.006	0.003	0.014	2.9	0.025	5.8	0.003	0.003	0.002	0.007	1.4	0.011	2.6
Chile	0.007	0.003	0.002	0.014	3.0	0.023	5.9	0.004	0.002	0.001	0.008	1.8	0.013	3.3
Colombia	0.011	0.008	0.005	0.010	2.0	0.018	3.6	0.010	0.006	0.004	0.007	1.4	0.013	2.5
Costa Rica	0.002	0.001	0	0.001	0.2	0.002	0.4	0.001	0	0	0.001	0.2	0.001	0.2
Dominican Republic	0.014	0.004	0.002	0.011	2.6	0.017	5.1	0.013	0.003	0.002	0.008	1.9	0.012	3.6
Ecuador	0.014	0.004	0.002	0.006	1.4	0.007	1.6	0.012	0.003	0.001	0.004	0.8	0.004	0.9
El Salvador	0.032	0.011	0.006	0.018	4.7	0.022	8.4	0.025	0.007	0.004	0.012	3.2	0.015	5.7
Guatemala	0.060	0.021	0.013	0.013	2.6	0.014	3.0	0.023	0.009	0.005	0.004	0.8	0.002	0.4
Jamaica	0.002	0.004	0.004	0.005	0.9	0.009	1.7	0	0.002	0.002	0.003	0.6	0.006	1.1
Mexico	0.017	0.008	0.005	0.008	1.6	0.010	1.8	0.009	0.004	0.003	0.004	0.8	0.004	0.8
Nicaragua	0	0	0	0	0	0	0							
Paraguay	0.005	0.003	0.002	0.006	1.2	0.007	1.4	0.001	0.002	0.001	0.003	0.7	0.003	0.7
Peru	0.033	0.017	0.012	0.014	2.8	0.018	3.3	0.019	0.011	0.007	0.008	1.6	0.010	1.8
Uruguay	0.001	0	0	0.002	0.4	0.003	0.7	0.001	0	0	0.001	0.2	0.002	0.5
Venezuela	0.005	0.002	0.001	0.005	1.4	0.005	2.5	0.003	0.001	0.001	0.001	0.2	0.0004	0.2

Source: Harmonised Household Surveys of Latin America and the Caribbean and Oxford Stringency Index and Google Workplace Mobility Data, authors’ own calculations.

Notes: The table reports the changes between the pre-lockdown situation and the situation at the end of the first phase of the lockdown assuming perfect compliance (columns 1 to 7) and imperfect compliance (columns 8 to 14). FGT_0_ is the headcount ratio. It is the fraction of workers with a labour income below the international poverty line of $5 (PPP). FGT_1_ is the average poverty gap and FGT_2_ measures the intensity/severity of poverty. G is the Gini coefficient and MLD is the mean logarithmic deviation index. $${\Delta }^{A}$$ is the absolute change in each measure while $${\Delta }^{R}$$ is the relative change in each measure (%). For the sake of simplicity, we do not report the measures for the pre-lockdown period as well as for the period at the end of the first phase of the lockdown. However, the results are available upon request. The sample for Argentina and the Bahamas is restricted only to urban areas. Therefore, the results for these two countries are not representative of the changes at the national level. The LAC changes are calculated as a weighted average of the population in all countries, excluding Argentina and the Bahamas, which are not representative at the national level, and Nicaragua to be consistent with the scenario under imperfect compliance.

^a^ Sample restricted to urban areas.

We also find that labour income inequality increases for the Gini coefficient and the MLD in almost all the LAC countries. The average increase in labour income inequality at the level of the LAC region is higher under perfect compliance (1.2 pp increase for the Gini coefficient and 2.1 pp for the MLD index) compared to under imperfect compliance (0.6 pp increase for the Gini coefficient and 1 pp increase for the MLD index). Across countries, the highest increase in the Gini coefficient under perfect compliance is observed in El Salvador with a 1.8 pp increase. This increase is reduced to 1.2 pp under imperfect compliance. Lastly, the highest increase in the MLD index under perfect compliance is in Brazil with a 2.5 pp increase. However, due to the high level of non-compliance in Brazil, this is no longer the case under imperfect compliance. The highest increase in the MLD index observed under de facto compliance is in El Salvador with a 1.5 pp increase. Overall, the inclusion of non-compliance attenuates the increase in poverty and inequality, and this effect is higher in countries with a higher level of non-compliance.

We decompose overall inequality into a between-countries and a within-countries component (Table 2). Our between-countries and within-countries components for the pre-lockdown period are consistent with what have been found previously in the literature (Ravallion and Chen 2012). Considering the changes between the two periods, our results show that social distancing has led to an increase in both inequality between and within countries. Yet, the between-countries inequality component increases significantly more than the within-countries inequality component. One explanation to this increase in the between-countries inequality component is that countries with lower levels of development have experienced relatively larger changes in their labour income distribution (due partly to a lower teleworking capacity), thus increasing inequality between countries.^36^ Similarly, the within-countries inequality component has increased due to social distancing since the lower part of the labour income distribution (mostly represented by socioeconomic vulnerable workers) has been the most affected in most of the LAC countries, exacerbating even further existing inequalities within countries.

**Table 2:** The between- and within-countries inequality components in Latin America and the Caribbean

	Gini	MLD	${MLD}^{BT}$	%	${MLD}^{WT}$	%
Under perfect compliance						
Baseline	0.49	0.48	0.019	4.0	0.461	96.0
	(0.001)	(0.002)	(0.0004)		(0.002)	
Lockdown	0.50	0.50	0.023	4.6	0.478	95.4
	(0.001)	(0.002)	(0.0004)		(0.002)	
∆^*A*^	0.012	0.021	0.004		0.017	
∆^*R*^ (%)	2.5	4.3	19.8		3.7	
Under imperfect compliance						
Baseline	0.49	0.48	0.019	4.0	0.461	96.0
	(0.001)	(0.002)	(0.0004)		(0.002)	
Lockdown	0.50	0.49	0.021	4.3	0.469	95.7
	(0.001)	(0.002)	(0.0004)		(0.005)	
∆^*A*^	0.006	0.010	0.002		0.008	
∆^*R*^ (%)	1.3	2.1	9.9		1.8	

Source: Harmonised Household Surveys of Latin America and the Caribbean and Oxford Stringency Index and Google Workplace Mobility Data, authors’ own calculations.

Notes: We apply the PPP (2011 USD) conversion factor to the total labour income in each country. ${\Delta }^{A}$ is the absolute change in labour income inequality while ${\Delta }^{R}$ is the relative change in labour income inequality (%). The LAC changes are calculated as a weighted average of the population in all countries, excluding Argentina and the Bahamas, which are not representative at the national level, and Nicaragua to be consistent with the scenario under imperfect compliance. Bootstrapped standard errors are reported in parentheses. Numbers have been rounded.

The fact that inequality between countries increases more than inequality within countries underlines the fact that the main changes in labour income inequality due to social distancing have happened between LAC countries.

When non-compliance is taken into account, our results on the decomposition of overall inequality differ in magnitude. Indeed, there is a smaller increase in both inequality between and within countries. This is not a surprising result since, within countries, the potential non-compliers are more likely to be among the poorest individuals, thus reducing the increase in inequality. Similarly, the countries that have lower levels of compliance are, in general, also the countries with lower levels of development and weaker institutionality (see Figure A.2 in Appendix A in the GLO discussion paper (GLO DP 682 [pre])), thus decreasing inequality between countries. Lastly, our results indicate that inequality in Latin America and the Caribbean is still largely explained by the within-countries component.

### Sources of labour income losses

The impact of the enforced social distancing measures on poverty and labour income inequality differs across LAC countries for a number of reasons. First, LAC countries have implemented different lockdown policies. Not all countries have implemented a lockdown. Among the countries that have implemented a lockdown, the social distancing policies differ in their duration and their strictness. This is likely to matter to explain labour income losses and changes in poverty and inequality. Second, the observed changes in poverty and labour income inequality depend on the structure of the economy that is observed. Since LAC countries differ in their sectoral/occupational employment structure, they do not experience similar changes. The countries that are characterised by a higher share of jobs that cannot be performed from home for instance are likely to experience a higher increase in poverty and labour income inequality. Lastly, we have seen that a higher level of non-compliance leads to a lower increase in poverty and inequality.

We focus on the role of the two first parameters, namely (i) the lockdown policy and (ii) conditional on the implementation of a lockdown, the sectoral/occupational employment structure of the economy in explaining the observed labour income losses and leave aside non-compliance to understand better the role of the first two components. More specifically, we conduct a series of counterfactual exercises under the scenario of perfect compliance. Our counterfactual exercise consists of assigning to all countries the same lockdown policy, which was implemented in the country that we select as the benchmark. We do this 19 times to subsequently select each country’s lockdown policy (in terms of strictness and duration) as the benchmark.^37^ The rationale behind this exercise is to eliminate cross-country differences in the lockdown policy and to observe the changes, knowing that the changes that are observed are now only due to the sectoral/occupational employment structure of the economies. Borrowing from Caselli (2005), we calculate a measure of the inter-percentile differential which can be expressed as follows:

7 $$\frac{{wl}_{benchmark}^{90}/{wl}_{benchmark}^{10}}{{wl}^{90}/{wl}^{10}}$$

This measure compares what the 90th to 10th percentile ratio would be in the counterfactual world with common lockdown policy, to the actual value. In other words, it calculates the dispersion of the labour income loss in all the countries under the same social distancing measures, the only difference left being the sectoral/occupational employment structure. This allows us to analyse the impact of each parameter separately: (i) the changes in labour income losses due to the lockdown policy and (ii) conditional on the implementation of a lockdown, the changes in labour income losses due to the sectoral/occupational employment structure of the economy.

We compute the inter-percentile ratio using wage losses adjusted by the purchasing power parity (PPP 2011 USD) factor conversion from local currency units to international dollars. The values we get for the inter-percentile ratio range from 70 to 84%, the simple average being 75%.^38^ This means that, conditional on applying a lockdown, the fraction of the cross-country labour income loss dispersion explained by the sectoral/occupational employment structure of LAC countries is, on average, 75%. In our framework, the rest would be explained by the type of lockdown (duration and strictness) that was implemented. This result highlights the importance of considering the sectoral/occupational employment structure of the country when implementing lockdowns, as this is a key factor in determining the magnitude and dispersion of potential labour income losses and therefore the impacts on poverty and labour income inequality.

## Conclusion

To prevent the spread of COVID-19, countries around the world have put in place broad social distancing policies. One of the implications is that many individuals have been unable to work during the lockdown. This study sheds light on the potential distributional consequences of the first phase of the lockdowns on poverty and labour income inequality in 20 Latin American and Caribbean countries. Besides, this study provides an informative comparison of the effects between de jure and de facto lockdowns. While the former assumes perfect compliance, de facto lockdowns are characterised by some degree of non-compliance.

Our results show a sizeable potential increase in poverty in almost all LAC countries. Under perfect compliance, the highest increase in the headcount poverty index is observed in Guatemala with a 6 pp increase. We also find that labour income inequality increases for the Gini coefficient and the MLD in almost all countries. The highest increase in the Gini coefficient is observed in El Salvador, reaching a 1.8 pp increase. Similarly, the highest increase in the MLD index is in Brazil with a 2.5 pp increase. The changes in poverty and labour income inequality are still positive when examining de facto compliance. However, the changes have been reduced, thus revealing the potential role of non-compliance in LAC countries as a coping strategy during the lockdowns.

Our results also highlight that lockdown measures are likely to worsen inequality in Latin America and the Caribbean both between and within countries. Our decomposition exercise shows that between-countries inequality increases by 19.8% under perfect compliance (9.9% under imperfect compliance) and within-countries inequality increases by 3.7% under perfect compliance (1.8% under imperfect compliance). The observed increase in between-countries inequality is due to the fact that countries with lower levels of development have been hit relatively harder. This is still the case under imperfect compliance even though it is a lower increase since poorer countries have lower levels of compliance. Similarly, within-country inequality increases since the lower part of the labour income distribution has been affected the most in most of the countries. This increase is attenuated under imperfect compliance though since the potential non-compliers are more likely to be among the most vulnerable individuals.

A number of factors can explain these observed differences in the changes in poverty and inequality across countries. First, the sectoral employment structure of each country plays an important role in explaining the impact of the shock on employment equilibria. Indeed, these differences in sectoral employment structures lead to differences in teleworking capacity. Countries with higher shares of teleworkability are better prepared to affront lockdowns and workers are relatively more protected against unemployment and labour income drops. These differences in terms of teleworking capacity across countries can be exacerbated by technological change: over the period of the pandemic, countries have probably shifted towards higher teleworking capacity.

Yet, additional factors matter to explain why the impact of the pandemic differs across countries. Our results indicate a different mapping of the shock under imperfect compliance compared to the situation in which there is perfect compliance. The level of development, level of informality in labour markets and government effectiveness are factors that can explain this observed cross-country heterogeneity. However, we acknowledge that our partial approach does not capture the full set of mappings that this exogenous shock has had in the labour markets; in particular, we assume that there are no changes in the demand for labour not related with the lockdowns. Yet, there might be other potential factors influencing the full set of new equilibria.

## Data Availability

The data collected from the laws and decrees is available upon request. However, the household survey data is not as the access is restricted.

## Appendix

Tables 3, 4, 5, 6, 7, 8, 9, 10, 11, 12, 13, 14, 15, 16, 17, 18 and 19.

**Table 3:** Individual-level data sources

Country	Country code	Survey year	Survey name	Sample size
Argentina^a^	ARG	2019	Encuesta Permanente de Hogares – Continua (EPHC)	115,748
Bahamas^a^	BHS	2014	Labour Force Survey (LFS)	6705
Barbados	BRB	2015	Continuous Labour Force Sample Survey (CLFSS)	13,579
Belize	BLZ	2007	Labour Force Survey (LFS)	8940
Bolivia	BOL	2018	Encuesta de Hogares (ECH)	37,517
Brazil	BRA	2015	Pesquisa Nacional por Amostra de Domicilio (PNAD)	355,935
Chile	CHL	2017	Encuesta de Caracterización Socioeconómica Nacional (CASEN)	216,439
Colombia	COL	2018	Gran Encuesta Integrada de Hogares (GEIH)	191,041
Costa Rica	CRI	2013	Encuesta Nacional de Hogares (ENAHO)	38,779
Dominican Republic	DOM	2019	Encuesta Nacional de Fuerza de Trabajo (ENFT)	20,965
Ecuador	ECU	2019	Encuesta Nacional de Empleo, Desempleo y Subempleo (ENEMDU)	59,208
El Salvador	SLV	2019	Encuesta de Hogares de Propósitios Múltiples (EHPM)	74,448
Guatemala	GTM	2019	Encuesta Nacional de Empleo e Ingresos (ENEI)	22,097
Jamaica	JAM	2014	Labour Force Survey (LFS)	20,444
Mexico	MEX	2018	Encuesta Nacional de Ingresos y Gastos de los Hogares (ENIGH)	269,206
Nicaragua	NIC	2014	Encuesta de Hogares sobre medición de Niveles de Vida (EMNV)	29,381
Paraguay	PRY	2017	Encuesta Permanente de Hogares (EPH)	35,215
Peru	PER	2019	Encuesta Nacional de Hogares (ENAHO)	124,979
Uruguay	URY	2019	Encuesta Continua de Hogares (ECH)	107,871
Venezuela	VEN	2015	Encuesta de Hogares por Muestreo (EHM)	117,919

Source: Harmonised Household Surveys of Latin America and the Caribbean, authors’ own calculations

Notes: For each country, we selected the latest harmonised survey available, except for Costa Rica for which we selected the year 2013. The reason for this is that this survey provides a variable at the 4-digit level needed to construct our standardised 2-digit ISIC classification. From 2014 onwards, this variable does not appear in the survey

^a^ For Argentina and the Bahamas, the sample is restricted to urban areas, while the other countries have national representativeness

**Table 4:** Share of individuals able to work from home, by 1-digit ISCO and by country

Countries	ISCO 1	ISCO 2	ISCO 3	ISCO 4	ISCO 5	ISCO 6	ISCO 7	ISCO 8	ISCO 9
All	0.29	0.31	0.26	0.45	0.07	0	0.03	0.003	0.009
Argentina ^a^	0.24	0.28	0.24	0.44	0.07	0	0.01	0.003	0.008
Bahamas ^a^	0.25	0.35	0.21	0.43	0.06	0	0.02	0.001	0.03
Barbados	0.31	0.28	0.12	0.48	0.04	0	0.02	0.001	0.006
Belize	0.37	0.27	0.27	0.45	0.09	0	0.02	0.001	0.01
Bolivia	0.24	0.28	0.25	0.45	0.07	0	0.03	0.001	0.02
Brazil	0.30	0.30	0.25	0.45	0.06	0	0.02	0.003	0.006
Chile	0.19	0.34	0.27	0.44	0.07	0	0.02	0.001	0.01
Colombia	0.44	0.43	0.28	0.49	0.07	0	0.03	0.005	0.01
Costa Rica	0.28	0.33	0.29	0.45	0.09	0	0.03	0.002	0.008
Dominican Republic	0.23	0.31	0.28	0.48	0.08	0	0.02	0.002	0.007
Ecuador	0.28	0.31	0.28	0.43	0.08	0	0.03	0.002	0.01
El Salvador	0.28	0.35	0.24	0.44	0.08	0	0.03	0.003	0.009
Guatemala	0.21	0.26	0.20	0.44	0.08	0	0.05	0.002	0.01
Jamaica	0.19	0.32	0.31	0.47	0.09	0	0.02	0.001	0.01
Mexico	0.23	0.31	0.27	0.46	0.08	0	0.04	0.002	0.009
Nicaragua	0.23	0.30	0.29	0.45	0.07	0	0.04	0.003	0.01
Paraguay	0.39	0.28	0.31	0.46	0.08	0	0.03	0.001	0.01
Peru	0.32	0.29	0.24	0.43	0.08	0	0.02	0.002	0.006
Uruguay	0.32	0.30	0.25	0.42	0.08	0	0.02	0.002	0.01
Venezuela	0.30	0.25	0.33	0.40	0.09	0	0.01	0.002	0.004

Source: Harmonised Household Surveys of Latin America and the Caribbean, authors’ own calculations

Notes: *ISCO 1* refers to “Managers”, *ISCO 2* refers to “Professionals”, *ISCO 3* refers to “Technicians and associate professionals”, *ISCO 4* refers to “Clerical support workers”, *ISCO 5* refers to “Service and sales workers”, *ISCO 6* refers to “Skilled agricultural, forestry and fishery workers”, *ISCO 7* refers to “Craft and related trades workers”, *ISCO 8* refers to “Plant and machine operators, and assemblers” and *ISCO 9* refers to “Elementary occupations”. The shares for the entire LAC region have been calculated as a weighted average of the population in each country, excluding Argentina and the Bahamas, which are not representative at the national level. When the information was not available, we leave the cells as empty

^a^ Sample restricted to urban areas

**Table 5:** Share of individuals able to work from home, by economic activity and by country

Countries	Activity 1	Activity 2	Activity 3	Activity 4	Activity 5	Activity 6	Activity 7	Activity 8	Activity 9
All	0.007	0.11	0.10	0.17	0.05	0.11	0.08	0.31	0.18
Argentina ^a^	0.11	0.19	0.10	0.16	0.04	0.11	0.07	0.29	0.17
Bahamas ^a^	0.10		0.06	0.03	0.06	0.11	0.04	0.15	0.12
Barbados									
Belize	0.01	0.08	0.09	0.11	0.07	0.14	0.16	0.27	0.16
Bolivia	0.004	0.15	0.08	0.13	0.03	0.08	0.02	0.37	0.20
Brazil	0.005	0.12	0.12	0.17	0.09	0.13	0.09	0.34	0.21
Chile	0.03	0.12	0.10	0.16	0.07	0.12	0.11	0.31	0.18
Colombia	0.02	0.07	0.10	0.20	0.15	0.09	0.08	0.21	0.22
Costa Rica	0.02	0.003	0.12	0.19	0.06	0.12	0.09	0.34	0.19
Dominican Republic	0.005	0.11	0.07	0.14	0.03	0.08	0.07	0.39	0.19
Ecuador	0.006	0.14	0.09	0.13	0.03	0.07	0.04	0.28	0.16
El Salvador	0.008	0.07	0.06	0.11	0.04	0.07	0.08	0.30	0.13
Guatemala	0.006	0.03	0.09	0.09	0.02	0.08	0.06	0.32	0.16
Jamaica	0.002	0.13	0.11	0.18	0.03	0.13	0.14	0.35	0.17
Mexico	0.007	0.10	0.08	0.17	0.04	0.13	0.06	0.37	0.15
Nicaragua	0.002	0.07	0.07	0.12	0.04	0.08	0.10	0.39	0.15
Paraguay	0.005	0.02	0.09	0.22	0.05	0.10	0.12	0.36	0.17
Peru	0.002	0.09	0.08	0.17	0.05	0.08	0.07	0.37	0.19
Uruguay	0.02	0.10	0.10	0.15	0.03	0.11	0.13	0.27	0.17
Venezuela	0.01	0.16	0.09	0.17	0.04	0.09	0.05	0.29	0.16

Source: Harmonised Household Surveys of Latin America and the Caribbean, authors’ own calculations

Notes: *Activity 1* refers to “Agriculture, hunting, forestry and fishing”, *activity 2* refers to “Mining and quarrying”, *activity 3* refers to “Manufacturing industries”, *activity 4* refers to “Electricity, gas and water”, *activity 5* refers to “Construction”, *activity 6* refers to “Wholesale and retail trade”, *activity 7* refers to “Transport and storage”, *activity 8* refers to “Financial insurance and real estate” and *activity 9* refers to “Social and community services”. The shares for the entire LAC region have been calculated as a weighted average of the population in each country, excluding Argentina and the Bahamas, which are not representative at the national level. When the information was not available, we leave the cells as empty

^a^ Sample restricted to urban areas

**Table 6:** Share of individuals able to work from home, by individual characteristics and by country

Countries	Male	Female	Below 9 years of education	Above 9 years of education	Rural	Urban	Informal	Formal	Size firm—small	Size firm—medium	size firm—large	Quintile total labour income—first	quintile total labour income—second	Quintile total labour income—third	Quintile total labour income—fourth	Quintile total labour income—fifth
All	0.10	0.15	0.05	0.18	0.05	0.14	0.07	0.18	0.08	0.15	0.18	0.07	0.09	0.11	0.14	0.21
Argentina ^a^	0.13	0.16	0.04	0.18		0.14	0.10	0.20	0.10	0.18	0.21	0.06	0.10	0.14	0.19	0.24
Bahamas ^a^	0.07	0.15				0.10	0.11	0.10	0.09			0.09	0.08	0.10	0.11	0.14
Barbados	0.10	0.21	0.05	0.17								0.10	0.11	0.15	0.21	0.27
Belize	0.09	0.16	0.06	0.21	0.07	0.16						0.10	0.12	0.11	0.11	0.10
Bolivia	0.08	0.11	0.03	0.13	0.03	0.11	0.05	0.21	0.05	0.18	0.20	0.03	0.07	0.09	0.12	0.15
Brazil	0.13	0.17	0.06	0.21	0.05	0.16	0.08	0.18	0.12	0.17	0.19	0.09	0.13	0.15	0.16	0.23
Chile	0.11	0.16	0.04	0.16	0.06	0.14	0.08	0.15	0.06	0.11	0.15	0.07	0.08	0.11	0.15	0.25
Colombia	0.11	0.15	0.04	0.18	0.04	0.15	0.07	0.21	0.07	0.17	0.23	0.05	0.06	0.12	0.15	0.26
Costa Rica	0.12	0.16	0.05	0.23	0.08	0.17	0.07	0.17	0.07	0.16	0.20	0.04	0.07	0.11	0.20	0.26
Dominican Republic	0.09	0.15	0.05	0.16	0.07	0.12	0.06	0.18	0.08	0.16	0.17	0.07	0.10	0.10	0.12	0.19
Ecuador	0.07	0.11	0.02	0.14	0.03	0.11	0.04	0.15	0.05	0.11	0.19	0.03	0.04	0.06	0.10	0.20
El Salvador	0.08	0.09	0.04	0.15	0.05	0.11	0.05	0.17	0.04	0.10	0.18	0.04	0.04	0.08	0.11	0.19
Guatemala	0.06	0.11	0.04	0.17	0.06	0.09	0.05	0.18	0.05	0.12	0.15	0.03	0.03	0.05	0.10	0.16
Jamaica	0.10	0.18	0.05	0.18	0.08	0.18			0.05	0.21	0.26	0.09	0.14	0.20	0.22	0.31
Mexico	0.09	0.12	0.05	0.18	0.05	0.12	0.07	0.17	0.06	0.15	0.15	0.06	0.07	0.08	0.12	0.19
Nicaragua	0.06	0.10	0.03	0.16	0.03	0.10	0.04	0.17				0.03	0.04	0.05	0.11	0.15
Paraguay	0.09	0.12	0.04	0.17	0.05	0.13	0.08	0.20	0.07	0.17	0.19	0.04	0.05	0.10	0.13	0.20
Peru	0.08	0.10	0.02	0.13	0.02	0.11	0.06	0.20	0.05	0.13	0.20	0.03	0.05	0.09	0.11	0.19
Uruguay	0.11	0.16	0.05	0.21	0.07	0.15	0.05	0.16	0.08	0.14	0.20	0.06	0.09	0.13	0.17	0.24
Venezuela	0.09	0.15	0.05	0.15			0.06	0.18	0.06	0.12	0.18	0.08	0.11	0.12	0.14	0.13

Source: Harmonised Household Surveys of Latin America and the Caribbean, authors’ own calculations

Notes: The shares for the entire LAC region have been calculated as a weighted average of the population in each country, excluding Argentina and the Bahamas, which are not representative at the national level. When the information was not available, we leave the cells as empty

^a^ Sample restricted to urban areas

**Table 7:** Share of individuals able to work under the lockdown with perfect compliance, by 1-digit ISCO and by country

Countries	ISCO 1	ISCO 2	ISCO 3	ISCO 4	ISCO 5	ISCO 6	ISCO 7	ISCO 8	ISCO 9
All	0.56	0.82	0.63	0.72	0.43	0.97	0.23	0.42	0.42
Argentina ^a^	0.35	0.82	0.59	0.63	0.24	0.82	0.11	0.18	0.19
Bahamas ^a^	0.86	0.67	0.48	0.43	0.47	0.50	0.52	0.60	0.67
Barbados	0.57	0.71	0.63	0.75	0.46	0.59	0.16	0.65	0.35
Belize	0.75	0.84	0.64	0.62	0.50	0.95	0.73	0.82	0.57
Bolivia	0.55	0.90	0.63	0.70	0.68	0.83	0.52	0.15	0.41
Brazil	0.46	0.83	0.66	0.71	0.29	0.99	0.22	0.52	0.57
Chile	0.67	0.84	0.72	0.72	0.65	0.87	0.21	0.32	0.50
Colombia	0.77	0.77	0.69	0.79	0.51	0.97	0.12	0.54	0.58
Costa Rica	0.91	0.98	0.94	0.93	0.83	1	0.97	0.98	0.93
Dominican Republic	0.56	0.85	0.68	0.50	0.59	0.95	0.15	0.18	0.39
Ecuador	0.66	0.88	0.68	0.73	0.63	0.99	0.18	0.19	0.33
El Salvador	0.58	0.79	0.64	0.63	0.21	0.99	0.30	0.18	0.41
Guatemala	0.80	0.84	0.60	0.66	0.66	0.96	0.24	0.15	0.40
Jamaica	0.73	0.81	0.69	0.75	0.66	0.95	0.18	0.88	0.48
Mexico	0.61	0.76	0.59	0.74	0.60	0.95	0.26	0.46	0.35
Nicaragua	1	1	1	1	1	1	1	1	1
Paraguay	0.69	0.86	0.63	0.69	0.72	0.95	0.15	0.65	0.31
Peru	0.73	0.86	0.44	0.65	0.15	1	0.17	0.10	0.34
Uruguay	0.89	0.96	0.92	0.94	0.88	1	0.98	0.98	0.94
Venezuela	0.63	0.82	0.58	0.76	0.73	0.96	0.13	0.15	0.43

Source: Harmonised Household Surveys of Latin America and the Caribbean, authors’ own calculations

Notes: *ISCO 1* refers to “Managers”, *ISCO 2* refers to “Professionals”, *ISCO 3* refers to “Technicians and associate professionals”, *ISCO 4* refers to “Clerical support workers”, *ISCO 5* refers to “Service and sales workers”, *ISCO 6* refers to “Skilled agricultural, forestry and fishery workers”, *ISCO 7* refers to “Craft and related trades workers”, *ISCO 8* refers to “Plant and machine operators, and assemblers” and *ISCO 9* refers to “Elementary occupations”. The shares for the entire LAC region have been calculated as a weighted average of the population in each country, excluding Argentina and the Bahamas, which are not representative at the national level, and Nicaragua to be consistent with the scenario under imperfect compliance. When the information was not available, we leave the cells as empty

^a^ Sample restricted to urban areas

**Table 8:** Share of individuals able to work under the lockdown with perfect compliance, by economic activity and by country

Countries	Activity 1	Activity 2	Activity 3	Activity 4	Activity 5	Activity 6	Activity 7	Activity 8	Activity 9
All	1	0.88	0.36	0.99	0.11	0.45	0.65	0.65	0.54
Argentina ^a^	1	1	0.39	1	0.04	0.06	0.07	0.43	0.63
Bahamas ^a^	1		0.29	1	0.46	0.34	0	0.79	0.74
Barbados									
Belize	1	1	1	1	1	0.46	0.84	0.55	0.44
Bolivia	0.82	1	0.35	1	1	0.57	0.007	0.83	0.67
Brazil	1	1	0.34	0.98	0.09	0.24	0.89	0.64	0.61
Chile	1	1	0.42	1	0.07	0.63	0.16	0.71	0.64
Colombia	1	1	0.46	1	0.15	0.46	0.99	0.52	0.54
Costa Rica	1	1	1	1	1	0.79	0.96	1	0.93
Dominican Republic	1	1	0.32	1	0.03	0.69	0.08	0.94	0.50
Ecuador	1	1	0.36	1	0.03	0.58	0.11	0.44	0.61
El Salvador	1	0	0.42	1	0.04	0.10	0.15	0.64	0.47
Guatemala	1	0.03	0.42	1	0.02	0.63	0.14	0.76	0.45
Jamaica	1	1	0.40	1	0.03	0.57	0.97	0.77	0.59
Mexico	1	0.24	0.32	1	0.04	0.81	0.98	1	0.35
Nicaragua	1	1	1	1	1	1	1	1	1
Paraguay	1	1	0.35	1	0.05	0.59	0.98	0.36	0.49
Peru	1	1	0.34	1	0.05	0.06	0.07	0.87	0.61
Uruguay	1	1	1	1	1	0.82	0.99	1	0.93
Venezuela	1	1	0.33	1	0.04	0.75	0.06	0.45	0.66

Source: Harmonised Household Surveys of Latin America and the Caribbean, authors’ own calculations

Notes: *Activity 1* refers to “Agriculture, hunting, forestry and fishing”, *activity 2* refers to “Mining and quarrying”, *activity 3* refers to “Manufacturing industries”, *activity 4* refers to “Electricity, gas and water”, *activity 5* refers to “Construction”, *activity 6* refers to “Wholesale and retail trade”, *activity 7* refers to “Transport and storage”, *activity 8* refers to “Financial insurance and real estate” and *activity 9* refers to “Social and community services”. The shares for the entire LAC region have been calculated as a weighted average of the population in each country, excluding Argentina and the Bahamas, which are not representative at the national level, and Nicaragua to be consistent with the scenario under imperfect compliance. When the information was not available, we leave the cells as empty

^a^ Sample restricted to urban areas

**Table 9:** Share of individuals able to work under the lockdown with perfect compliance, by individual characteristics and by country

Countries	Male	Female	Below 9 years of education	Above 9 years of education	Rural	Urban	Informal	Formal	Size firm—small	Size firm—medium	Size firm—large	Quintile total labour income—first	Quintile total labour income—second	Quintile total labour income—third	Quintile total labour income—fourth	Quintile total labour income—fifth
All	0.56	0.53	0.52	0.57	0.72	0.51	0.50	0.60	0.49	0.52	0.60	0.55	0.50	0.51	0.53	0.63
Argentina ^a^	0.32	0.44	0.19	0.44		0.37	0.21	0.56	0.20	0.44	0.67	0.19	0.28	0.38	0.49	0.59
Bahamas ^a^	0.51	0.55				0.53	0.49	0.54	0.55			0.49	0.56	0.48	0.53	0.66
Barbados	0.47	0.57	0.40	0.53								0.41	0.45	0.48	0.66	0.68
Belize	0.75	0.56	0.69	0.66	0.75	0.62						0.69	0.69	0.67	0.63	0.68
Bolivia	0.61	0.63	0.63	0.61	0.76	0.57	0.56	0.80	0.58	0.73	0.83	0.67	0.55	0.58	0.64	0.64
Brazil	0.58	0.49	0.49	0.57	0.77	0.51	0.47	0.57	0.40	0.35	0.52	0.50	0.46	0.52	0.55	0.66
Chile	0.52	0.65	0.51	0.59	0.70	0.56	0.49	0.61	0.39	0.50	0.61	0.52	0.55	0.55	0.56	0.69
Colombia	0.66	0.54	0.59	0.62	0.80	0.56	0.56	0.68	0.56	0.51	0.76	0.56	0.56	0.58	0.61	0.73
Costa Rica	0.94	0.90	0.92	0.93	0.93	0.92	0.91	0.93	0.92	0.87	0.95	0.92	0.91	0.91	0.92	0.95
Dominican Republic	0.46	0.51	0.42	0.51	0.57	0.46	0.37	0.62	0.41	0.58	0.65	0.42	0.43	0.48	0.44	0.56
Ecuador	0.56	0.62	0.59	0.58	0.78	0.51	0.53	0.67	0.53	0.57	0.79	0.65	0.53	0.50	0.51	0.70
El Salvador	0.48	0.30	0.39	0.43	0.51	0.35	0.37	0.51	0.39	0.43	0.60	0.31	0.37	0.30	0.33	0.54
Guatemala	0.62	0.57	0.57	0.60	0.65	0.56	0.60	0.63	0.60	0.63	0.60	0.71	0.68	0.54	0.50	0.59
Jamaica	0.71	0.67	0.72	0.68	0.75	0.65			0.67	0.71	0.77	0.57	0.55	0.69	0.73	0.84
Mexico	0.52	0.55	0.50	0.58	0.67	0.49	0.53	0.54	0.53	0.55	0.50	0.61	0.50	0.45	0.48	0.60
Nicaragua	1	1	1	1	1	1	1	1				1	1	1	1	1
Paraguay	0.56	0.57	0.55	0.58	0.70	0.50	0.52	0.70	0.60	0.60	0.74	0.65	0.48	0.49	0.54	0.65
Peru	0.48	0.40	0.51	0.41	0.78	0.36	0.38	0.65	0.35	0.42	0.74	0.52	0.41	0.38	0.40	0.51
Uruguay	0.94	0.93	0.94	0.93	0.95	0.93	0.92	0.94	0.94	0.90	0.95	0.92	0.92	0.94	0.95	0.96
Venezuela	0.48	0.67	0.46	0.60			0.43	0.72	0.43	0.52	0.78	0.59	0.58	0.55	0.56	0.50

Source: Harmonised Household Surveys of Latin America and the Caribbean, authors’ own calculations

Notes: The shares for the entire LAC region have been calculated as a weighted average of the population in each country, excluding Argentina and the Bahamas, which are not representative at the national level, and Nicaragua to be consistent with the scenario under imperfect compliance. When the information was not available, we leave the cells as empty

^a^ Sample restricted to urban areas

**Table 10:** Percentage point variation in the share of individuals able to work under the lockdown due to imperfect compliance, by 1-digit ISCO and by country

Countries	ISCO 1	ISCO 2	ISCO 3	ISCO 4	ISCO 5	ISCO 6	ISCO 7	ISCO 8	ISCO 9
All	0.18	0.07	0.14	0.11	0.22	0.009	0.28	0.21	0.21
Argentina ^a^	0.18	0.05	0.11	0.10	0.21	0.06	0.24	0.22	0.22
Bahamas ^a^									
Barbados	0.04	0.03	0.04	0.03	0.05	0.04	0.08	0.04	0.06
Belize	0.06	0.04	0.08	0.08	0.11	0.01	0.06	0.04	0.09
Bolivia	0.09	0.02	0.08	0.06	0.07	0.04	0.10	0.18	0.13
Brazil	0.23	0.07	0.15	0.12	0.31	0.002	0.34	0.20	0.19
Chile	0.09	0.04	0.08	0.07	0.10	0.04	0.22	0.19	0.14
Colombia	0.05	0.05	0.07	0.05	0.12	0.008	0.21	0.11	0.10
Costa Rica	0.02	0.007	0.02	0.02	0.05	0	0.008	0.005	0.02
Dominican Republic	0.08	0.03	0.06	0.10	0.09	0.01	0.18	0.17	0.13
Ecuador	0.12	0.04	0.11	0.09	0.13	0.004	0.28	0.27	0.23
El Salvador	0.10	0.05	0.08	0.09	0.18	0.003	0.16	0.19	0.14
Guatemala	0.09	0.07	0.18	0.15	0.15	0.02	0.34	0.38	0.27
Jamaica	0.10	0.07	0.11	0.09	0.11	0.02	0.29	0.04	0.19
Mexico	0.15	0.09	0.16	0.10	0.16	0.02	0.29	0.22	0.26
Nicaragua									
Paraguay	0.11	0.05	0.13	0.11	0.10	0.02	0.31	0.13	0.25
Peru	0.10	0.05	0.18	0.11	0.28	0	0.27	0.29	0.21
Uruguay	0.03	0.01	0.03	0.02	0.04	0.002	0.005	0.008	0.02
Venezuela	0.15	0.07	0.17	0.10	0.11	0.01	0.36	0.35	0.24

Source: Harmonised Household Surveys of Latin America and the Caribbean and Oxford Stringency Index and Google Workplace Mobility Data, authors’ own calculations

Notes: *ISCO 1* refers to “Managers”, *ISCO 2* refers to “Professionals”, *ISCO 3* refers to “Technicians and associate professionals”, *ISCO 4* refers to “Clerical support workers”, *ISCO 5* refers to “Service and sales workers”, *ISCO 6* refers to “Skilled agricultural, forestry and fishery workers”, *ISCO 7* refers to “Craft and related trades workers”, *ISCO 8* refers to “Plant and machine operators, and assemblers” and *ISCO 9* refers to “Elementary occupations”. The shares for the entire LAC region have been calculated as a weighted average of the population in each country, excluding Argentina and the Bahamas, which are not representative at the national level, and Nicaragua to be consistent with the scenario under imperfect compliance. The Bahamas and Nicaragua are the two countries for which we are not able to conduct this exercise. For the Bahamas, we cannot calculate non-compliance; and for Nicaragua, there was no lockdown. For the other countries, when the information was not available, we leave the cells as empty

^a^ Sample restricted to urban areas

**Table 11:** Percentage point variation in the share of individuals able to work under the lockdown due to imperfect compliance, by economic activity and by country

Countries	Activity 1	Activity 2	Activity 3	Activity 4	Activity 5	Activity 6	Activity 7	Activity 8	Activity 9
All	0	0.04	0.24	0.002	0.31	0.21	0.12	0.13	0.17
Argentina ^a^	0	0	0.16	0	0.26	0.26	0.24	0.16	0.10
Bahamas ^a^									
Barbados									
Belize	0	0	0	0	0	0.12	0.04	0.10	0.12
Bolivia	0.04	0	0.14	0	0	0.09	0.21	0.04	0.07
Brazil	0	0	0.28	0.007	0.41	0.33	0.04	0.15	0.17
Chile	0	0	0.16	0	0.26	0.10	0.23	0.07	0.10
Colombia	0	0	0.13	0	0.21	0.13	0.002	0.11	0.11
Costa Rica	0	0	0	0	0	0.06	0.01	0	0.02
Dominican Republic	0	0	0.14	0	0.21	0.06	0.19	0.01	0.10
Ecuador	0	0	0.22	0	0.33	0.14	0.30	0.19	0.13
El Salvador	0	0.24	0.13	0	0.22	0.21	0.20	0.08	0.12
Guatemala	0	0.43	0.26	0	0.44	0.17	0.39	0.11	0.25
Jamaica	0	0	0.22	0	0.34	0.15	0.01	0.08	0.15
Mexico	0	0.30	0.28	0	0.38	0.08	0.006	0	0.25
Nicaragua									
Paraguay	0	0	0.24	0	0.35	0.15	0.005	0.22	0.19
Peru	0.001	0	0.22	0	0.31	0.30	0.30	0.04	0.13
Uruguay	0	0	0	0	0	0.06	0.002	0	0.02
Venezuela	0	0	0.27	0	0.39	0.10	0.38	0.23	0.14

Source: Harmonised Household Surveys of Latin America and the Caribbean and Oxford Stringency Index and Google Workplace Mobility Data, authors’ own calculations

Notes: *Activity 1* refers to “Agriculture, hunting, forestry and fishing”, *activity 2* refers to “Mining and quarrying”, *activity 3* refers to “Manufacturing industries”, *activity 4* refers to “Electricity, gas and water”, *activity 5* refers to “Construction”, *activity 6* refers to “Wholesale and retail trade”, *activity 7* refers to “Transport and storage”, *activity 8* refers to “Financial insurance and real estate” and *activity 9* refers to “Social and community services”. The shares for the entire LAC region have been calculated as a weighted average of the population in each country, excluding Argentina and the Bahamas, which are not representative at the national level, and Nicaragua to be consistent with the scenario under imperfect compliance. The Bahamas and Nicaragua are the two countries for which we are not able to conduct this exercise. For the Bahamas, we cannot calculate non-compliance; and for Nicaragua, there was no lockdown. For the other countries, when the information was not available, we leave the cells as empty

^a^ Sample restricted to urban areas

**Table 12:** Percentage point variation in the share of individuals able to work under the lockdown due to imperfect compliance, by individual characteristics and by country

Countries	Male	Female	Below 9 years of education	Above 9 years of education	Rural	Urban	Informal	Formal	Size firm—small	Size firm—medium	Size firm—large	Quintile total labour income—first	Quintile total labour income—second	Quintile total labour income—third	Quintile total labour income—fourth	Quintile total labour income—fifth
All	0.17	0.18	0.19	0.16	0.10	0.18	0.18	0.16	0.17	0.17	0.17	0.17	0.18	0.18	0.18	0.14
Argentina ^a^	0.18	0.15	0.22	0.15		0.17	0.21	0.12	0.22	0.16	0.09	0.23	0.20	0.17	0.14	0.11
Bahamas ^a^																
Barbados	0.05	0.04	0.06	0.05								0.06	0.06	0.05	0.03	0.03
Belize	0.06	0.10	0.07	0.08	0.05	0.09						0.07	0.07	0.08	0.09	0.07
Bolivia	0.08	0.08	0.08	0.08	0.05	0.09	0.09	0.04	0.09	0.06	0.04	0.07	0.09	0.09	0.08	0.08
Brazil	0.18	0.22	0.22	0.19	0.10	0.21	0.23	0.18	0.26	0.28	0.20	0.22	0.23	0.20	0.19	0.15
Chile	0.13	0.10	0.14	0.11	0.09	0.12	0.14	0.11	0.17	0.14	0.11	0.13	0.12	0.12	0.12	0.08
Colombia	0.08	0.11	0.10	0.09	0.05	0.10	0.10	0.08	0.10	0.11	0.06	0.11	0.10	0.10	0.09	0.06
Costa Rica	0.02	0.03	0.02	0.02	0.02	0.02	0.03	0.02	0.02	0.04	0.01	0.02	0.03	0.03	0.02	0.01
Dominican Republic	0.11	0.10	0.12	0.10	0.10	0.11	0.13	0.08	0.13	0.08	0.07	0.13	0.12	0.11	0.11	0.09
Ecuador	0.15	0.13	0.14	0.14	0.08	0.17	0.16	0.11	0.16	0.15	0.07	0.12	0.16	0.17	0.17	0.10
El Salvador	0.12	0.16	0.14	0.13	0.11	0.15	0.15	0.11	0.14	0.13	0.09	0.16	0.15	0.16	0.15	0.11
Guatemala	0.17	0.19	0.20	0.18	0.16	0.20	0.18	0.17	0.18	0.16	0.18	0.13	0.15	0.21	0.22	0.18
Jamaica	0.10	0.12	0.10	0.11	0.08	0.13			0.12	0.10	0.07	0.15	0.15	0.11	0.10	0.06
Mexico	0.19	0.18	0.20	0.16	0.13	0.20	0.18	0.18	0.18	0.18	0.20	0.15	0.20	0.22	0.21	0.16
Nicaragua																
Paraguay	0.16	0.16	0.16	0.15	0.12	0.18	0.18	0.10	0.15	0.15	0.09	0.13	0.19	0.18	0.16	0.12
Peru	0.17	0.19	0.16	0.19	0.07	0.21	0.20	0.12	0.21	0.19	0.09	0.15	0.19	0.20	0.20	0.16
Uruguay	0.02	0.02	0.02	0.02	0.01	0.02	0.03	0.02	0.02	0.03	0.01	0.03	0.02	0.02	0.02	0.01
Venezuela	0.21	0.14	0.22	0.16			0.23	0.12	0.24	0.20	0.09	0.17	0.17	0.19	0.18	0.21

Source: Harmonised Household Surveys of Latin America and the Caribbean and Oxford Stringency Index and Google Workplace Mobility Data, authors’ own calculations

Notes: The shares for the entire LAC region have been calculated as a weighted average of the population in each country, excluding Argentina and the Bahamas, which are not representative at the national level, and Nicaragua to be consistent with the scenario under imperfect compliance. The Bahamas and Nicaragua are the two countries for which we are not able to conduct this exercise. For the Bahamas, we cannot calculate non-compliance; and for Nicaragua, there was no lockdown. For the other countries, when the information was not available, we leave the cells as empty

^a^ Sample restricted to urban areas

**Table 13:** Social assistance programmes during COVID-19 pandemic, by country

Country	Programmes
Argentina	(1) Ingreso familiar de emergencia – transfer for households with a household head between 18 and 65 who works in domestic service, is an informal worker, is a monostributista social (categories A and B), or households receiving AUH or Progresar social programmes; household must not have a formal source of labour income or receive any pensions
Bahamas	(1) B$25 million for health care (2) B$5 million for food programmes (3) B$145 million for income support for job loss workers and self-employed (4) B$1.8 million to support to Family Islands (specifically to be used for any COVID-19–related expenditure)
Barbados	(1) Unemployment assistance for COVID-19 (2) Unemployment Programme for Self-Employed
Belize	(1) Providing short-term relief to employees affected by the crisis, especially those in the tourism sector (2) Additional support to the healthcare sector and the unemployed has been financed with loans from bilateral and multilateral creditors
Bolivia	(1) Direct relief payments to poorer households of about $US73 per child to households with children in public schools and students in private schools (2) Programme (Canasta Familiar) to make direct payments for food to 1.5 million families ($US58 per family) (3) Payments to electric bills for 3 months for the consumers with lower consumption and pay 50% of the potable water and gas for all households (4) The latest transfer to households (Bono Contra el Hambre) became available starting on December 2020. It provides a one-off transfer of about $146 for all eligible individuals
Brazil	(1) Transfer for households with individuals whose main source of income comes from being informal workers or self-employed; unemployed; or microentrepreneurs; these households must not be beneficiaries of Bolsa Familia; their total income must not be more than R$3135 or total per capita income above R$522.5
Chile	(1) Ingreso Familiar de Emergencia — transfer for households whose source of income is mainly from informal sources. The amount depends on the number of people in the household and decreases according to the percentage of income that is formal; pensioners from Pension Solidaria de la Vejez receive a smaller amount of aid (2) Bono de Emergencia COVID 19 — this transfer aims at households with individuals receiving Subsidio Familiar (SUF), households in the Sistema de Seguridades y Oportunidades database, households who belong to the 60% most vulnerable according to the Registro Social de Hogares database and households who do not have a formal income through employment or pension and do not have any SUF beneficiaries
Colombia	(1) Payments to health providers for ICU availability (2) Creation of a National Tracking and Contact Center (3) A one-off bonus for health workers (4) Delayed utility payments for poor and middle-income households (5) Transfers for vulnerable groups, and additional benefits for recently unemployed workers
Costa Rica	(1) A monthly subsidy of ¢125,000 (US$205) for 3 months to about 375 thousand households economically affected by the crisis with a monthly income of less than ¢750,000 (US$1230) prior to COVID-19 (2) An increase in public health spending, including construction of a specialized hospital for COVID-19 treatment and purchase of COVID-19 vaccines
Dominican Republic	(1) The *Quédate en Casa* programme, subsidizing the most vulnerable households, including informal workers (2) Coverage under the existing programme *Comer es Primero*, paying RD$5000 (roughly US$90) per month (3) 452,817 families receive additional transfers of RD$2000 (about US$36) per month (4) *Employee Solidarity Assistance Fund*, benefits about 754,000 families of formal workers who were laid off with a monthly transfer up to 70% of last formal wages (minimum of RD$5000, RD$8104 on average) (5) A programme *Pati* was introduced to support independent workers, providing RD$5000 (about US$90) a month to each beneficiary with an additional allowance made available for healthcare workers, the military and police officers, amounting to RD$2.4 billion
Ecuador	(1) Transfer for affiliates to the unpaid work regime or self-employed; or affiliates to the Seguro Social Campesino, with income lower than US$400 and who are not registered to the contributive social security and are not registered as dependents; individuals must not be beneficiaries of any other programmes of the government (2) Transfer for people not included in the previous subgroup whose income is lower than $400 and are below the poverty line
El Salvador	(1) Transfer for informal employees and self-employed workers with low social economic resources
Guatemala	(1) Electricity subsidies (2) Fostering low-income housing
Jamaica	(1) Temporary cash transfer to individuals for whom loss of employment can be verified since March 10 (2) Grants targeted at the most vulnerable segments of society
Mexico	(1) The government is providing subsidized unemployment insurance for 3 months to workers that hold a mortgage with the Housing Institute (5.9 billion pesos) (2) Additional resources are allocated to social spending related to infrastructure, security, education, urban improvement and other areas (62 billion pesos)
Nicaragua	(1) Provision of food packages among vulnerable families
Paraguay	(1) Supporting vulnerable population
Peru	(1) Bono Independiente — transfer for households with main income source coming from self-employment and not in poverty; households cannot be beneficiaries of the Juntos, Pension 65, or Contigo programmes; none of the household members can be registered as dependent workers of the public or private sector; household members cannot have income over PEN$1200 and cannot be part of any local or central government
Uruguay	(1) Transfer for food purchases for informal and self-employed workers, with no other social programme benefits and who do not have social security
Venezuela	NA

Source: Taken from Busso et al. (2020) and complemented with IMF-COVID tracker and other sources

**Table 14:** Mean loss labour income rate with perfect compliance, by 1-digit ISCO and by country

Countries	ISCO 1	ISCO 2	ISCO 3	ISCO 4	ISCO 5	ISCO 6	ISCO 7	ISCO 8	ISCO 9
All	0.08	0.04	0.07	0.06	0.12	0.005	0.15	0.12	0.12
Argentina ^a^	0.13	0.04	0.09	0.09	0.17	0.02	0.19	0.19	0.20
Bahamas ^a^	0.03	0.07	0.10	0.10	0.11	0.06	0.10	0.10	0.08
Barbados	0.02	0.01	0.02	0.01	0.03	0.02	0.04	0.02	0.03
Belize	0.03	0.02	0.04	0.05	0.06	0.006	0.03	0.02	0.06
Bolivia	0.09	0.02	0.07	0.06	0.06	0.03	0.09	0.16	0.11
Brazil	0.10	0.03	0.06	0.06	0.14	0	0.15	0.09	0.09
Chile	0.08	0.04	0.07	0.07	0.08	0.02	0.16	0.14	0.11
Colombia	0.04	0.04	0.06	0.04	0.09	0.006	0.16	0.08	0.08
Costa Rica	0.01	0.003	0.008	0.008	0.02	0	0.004	0.002	0.009
Dominican Republic	0.08	0.03	0.06	0.09	0.08	0.009	0.15	0.15	0.11
Ecuador	0.04	0.01	0.04	0.03	0.05	0.002	0.11	0.10	0.09
El Salvador	0.10	0.05	0.09	0.09	0.19	0.002	0.17	0.20	0.14
Guatemala	0.07	0.06	0.15	0.12	0.12	0.01	0.28	0.31	0.22
Jamaica	0.002	0.004	0.006	0.01	0.009	0.001	0.01	0.003	0.02
Mexico	0.07	0.04	0.08	0.05	0.07	0.009	0.14	0.10	0.12
Nicaragua	0	0	0	0	0	0	0	0	0
Paraguay	0.04	0.02	0.05	0.04	0.03	0.006	0.10	0.04	0.08
Peru	0.07	0.04	0.15	0.09	0.23	0	0.22	0.24	0.18
Uruguay	0.01	0.006	0.01	0.009	0.02	0	0.002	0.003	0.008
Venezuela	0.08	0.04	0.09	0.05	0.06	0.008	0.19	0.18	0.13

Source: Harmonised Household Surveys of Latin America and the Caribbean, authors’ own calculations

Notes: *ISCO 1* refers to “Managers”, *ISCO 2* refers to “Professionals”, *ISCO 3* refers to “Technicians and associate professionals”, *ISCO 4* refers to “Clerical support workers”, *ISCO 5* refers to “Service and sales workers”, *ISCO 6* refers to “Skilled agricultural, forestry and fishery workers”, *ISCO 7* refers to “Craft and related trades workers”, *ISCO 8* refers to “Plant and machine operators, and assemblers” and *ISCO 9* refers to “Elementary occupations”. The rates for the entire LAC region have been calculated as a weighted average of the population in each country, excluding Argentina and the Bahamas, which are not representative at the national level, and Nicaragua to be consistent with the scenario under imperfect compliance. When the information was not available, we leave the cells as empty

^a^ Sample restricted to urban areas

**Table 15:** Mean loss labour income rate with perfect compliance, by economic activity and by country

Countries	Activity 1	Activity 2	Activity 3	Activity 4	Activity 5	Activity 6	Activity 7	Activity 8	Activity 9
All	0	0.02	0.13	0.002	0.18	0.11	0.08	0.07	0.09
Argentina ^a^	0	0	0.15	0	0.21	0.20	0.21	0.13	0.09
Bahamas ^a^	0		0.17	0	0.09	0.14	0.19	0.02	0.05
Barbados									
Belize	0	0	0	0	0	0.07	0.02	0.05	0.07
Bolivia	0.03	0	0.12	0	0	0.08	0.19	0.03	0.06
Brazil	0	0	0.12	0.004	0.15	0.15	0.02	0.07	0.08
Chile	0	0	0.13	0	0.19	0.08	0.18	0.08	0.09
Colombia	0	0	0.10	0	0.16	0.10	0.002	0.09	0.08
Costa Rica	0	0	0	0	0	0.03	0.005	0	0.009
Dominican Republic	0	0	0.12	0	0.18	0.06	0.17	0.01	0.09
Ecuador	0	0	0.08	0	0.13	0.05	0.12	0.07	0.05
El Salvador	0	0.24	0.14	0	0.23	0.21	0.20	0.09	0.13
Guatemala	0	0.35	0.21	0	0.35	0.13	0.31	0.09	0.20
Jamaica	0	0	0.01	0	0.02	0.01	0.001	0.004	0.01
Mexico	0	0.14	0.13	0	0.18	0.04	0.003	0	0.12
Nicaragua	0	0	0	0	0	0	0	0	0
Paraguay	0	0	0.08	0	0.12	0.05	0.002	0.08	0.06
Peru	0.001	0	0.18	0	0.26	0.25	0.25	0.03	0.11
Uruguay	0	0	0	0	0	0.02	0	0	0.009
Venezuela	0	0	0.14	0	0.21	0.05	0.20	0.12	0.07

Source: Harmonised Household Surveys of Latin America and the Caribbean, authors’ own calculations

Notes: *Activity 1* refers to “Agriculture, hunting, forestry and fishing”, *activity 2* refers to “Mining and quarrying”, *activity 3* refers to “Manufacturing industries”, *activity 4* refers to “Electricity, gas and water”, *activity 5* refers to “Construction”, *activity 6* refers to “Wholesale and retail trade”, *activity 7* refers to “Transport and storage”, *activity 8* refers to “Financial insurance and real estate” and *activity 9* refers to “Social and community services”. The rates for the entire LAC region have been calculated as a weighted average of the population in each country, excluding Argentina and the Bahamas, which are not representative at the national level, and Nicaragua to be consistent with the scenario under imperfect compliance. When the information was not available, we leave the cells as empty

^a^ Sample restricted to urban areas

**Table 16:** Mean loss labour income rate with perfect compliance, by individual characteristics and by country

Countries	Male	Female	Below 9 years of education	Above 9 years of education	Rural	Urban	Informal	Formal	Size firm—small	Size firm—medium	Size firm—large	Quintile total labour income—first	Quintile total labour income—second	Quintile total labour income—third	Quintile total labour income—fourth	Quintile total labour income—fifth
All	0.09	0.09	0.10	0.09	0.06	0.10	0.10	0.08	0.11	0.10	0.08	0.09	0.10	0.10	0.10	0.08
Argentina ^a^	0.15	0.13	0.18	0.13		0.14	0.17	0.11	0.17	0.13	0.08	0.22	0.20	0.18	0.15	0.12
Bahamas ^a^	0.09	0.10				0.10	0.10	0.10	0.11			0.12	0.11	0.12	0.11	0.08
Barbados	0.03	0.02	0.03	0.02								0.03	0.03	0.03	0.02	0.02
Belize	0.03	0.06	0.04	0.04	0.03	0.05						0.05	0.05	0.05	0.06	0.05
Bolivia	0.07	0.07	0.07	0.07	0.05	0.08	0.08	0.04	0.08	0.05	0.03	0.06	0.09	0.08	0.07	0.07
Brazil	0.08	0.10	0.10	0.08	0.05	0.10	0.11	0.08	0.12	0.13	0.09	0.11	0.11	0.09	0.09	0.07
Chile	0.11	0.08	0.10	0.09	0.03	0.10	0.11	0.09	0.13	0.10	0.09	0.10	0.09	0.11	0.11	0.08
Colombia	0.06	0.08	0.08	0.07	0.04	0.08	0.08	0.06	0.08	0.09	0.05	0.08	0.08	0.08	0.07	0.05
Costa Rica	0.008	0.01	0.01	0.009	0.009	0.01	0.01	0.009	0.01	0.02	0.006	0.01	0.01	0.01	0.01	0.006
Dominican Republic	0.10	0.09	0.11	0.09	0.08	0.10	0.11	0.07	0.11	0.08	0.06	0.11	0.11	0.10	0.10	0.08
Ecuador	0.06	0.05	0.05	0.05	0.03	0.06	0.06	0.04	0.06	0.05	0.03	0.05	0.06	0.07	0.06	0.04
El Salvador	0.12	0.17	0.14	0.14	0.12	0.16	0.15	0.12	0.15	0.14	0.09	0.16	0.15	0.17	0.16	0.11
Guatemala	0.14	0.15	0.16	0.15	0.13	0.16	0.15	0.13	0.15	0.13	0.15	0.10	0.12	0.17	0.18	0.15
Jamaica	0.005	0.01	0.007	0.008	0.007	0.008			0.007	0.01	0.008	0.03	0.03	0.02	0.02	0.01
Mexico	0.09	0.08	0.09	0.08	0.06	0.10	0.09	0.09	0.09	0.08	0.09	0.07	0.09	0.10	0.10	0.08
Nicaragua	0	0	0	0	0	0	0	0				0	0	0	0	0
Paraguay	0.05	0.05	0.06	0.05	0.04	0.06	0.06	0.04	0.05	0.05	0.03	0.04	0.06	0.06	0.06	0.04
Peru	0.14	0.16	0.13	0.16	0.06	0.17	0.17	0.09	0.18	0.16	0.07	0.13	0.16	0.17	0.16	0.13
Uruguay	0.008	0.01	0.008	0.009	0.006	0.009	0.01	0.008	0.007	0.01	0.007	0.01	0.01	0.009	0.007	0.005
Venezuela	0.11	0.07	0.12	0.08			0.12	0.06	0.12	0.11	0.05	0.10	0.10	0.11	0.11	0.13

Source: Harmonised Household Surveys of Latin America and the Caribbean, authors’ own calculations

Notes: The rates for the entire LAC region have been calculated as a weighted average of the population in each country, excluding (i) Argentina and the Bahamas that are not representative at the national level and (ii) Nicaragua to be consistent with the scenario under imperfect compliance. When the information was not available, we leave the cells as empty

^a^ Sample restricted to urban areas

**Table 17:** Percentage point variation in the mean loss labour income rate due to imperfect compliance, by 1-digit ISCO and by country

Countries	ISCO 1	ISCO 2	ISCO 3	ISCO 4	ISCO 5	ISCO 6	ISCO 7	ISCO 8	ISCO 9
All	− 0.03	− 0.01	− 0.03	− 0.02	− 0.05	− 0.002	− 0.06	− 0.04	− 0.04
Argentina ^a^	− 0.03	− 0.01	− 0.03	− 0.02	− 0.05	− 0.007	− 0.05	− 0.05	− 0.05
Bahamas ^a^									
Barbados	− 0.002	− 0.001	− 0.002	− 0.001	− 0.003	− 0.002	− 0.004	− 0.002	− 0.003
Belize	− 0.006	− 0.004	− 0.009	− 0.01	− 0.01	− 0.001	− 0.007	− 0.005	− 0.01
Bolivia	− 0.02	− 0.004	− 0.01	− 0.01	− 0.01	− 0.007	− 0.02	− 0.03	− 0.02
Brazil	− 0.05	− 0.01	− 0.03	− 0.02	− 0.06	0	− 0.07	− 0.04	− 0.04
Chile	− 0.02	− 0.01	− 0.02	− 0.02	− 0.02	− 0.006	− 0.04	− 0.04	− 0.03
Colombia	− 0.01	− 0.01	− 0.01	− 0.009	− 0.02	− 0.001	− 0.04	− 0.02	− 0.02
Costa Rica	− 0.003	0	− 0.002	− 0.002	− 0.006	0	− 0.001	0	− 0.003
Dominican Republic	− 0.01	− 0.005	− 0.01	− 0.02	− 0.02	− 0.002	− 0.03	− 0.03	− 0.02
Ecuador	− 0.01	− 0.005	− 0.01	− 0.01	− 0.02	0	− 0.04	− 0.04	− 0.03
El Salvador	− 0.02	− 0.01	− 0.02	− 0.02	− 0.04	0	− 0.04	− 0.05	− 0.03
Guatemala	− 0.03	− 0.03	− 0.07	− 0.06	− 0.06	− 0.006	− 0.12	− 0.14	− 0.10
Jamaica	0	− 0.001	− 0.002	− 0.003	− 0.003	0	− 0.004	− 0.001	− 0.006
Mexico	− 0.03	− 0.02	− 0.03	− 0.02	− 0.03	− 0.004	− 0.05	− 0.04	− 0.05
Nicaragua									
Paraguay	− 0.01	− 0.006	− 0.02	− 0.01	− 0.01	− 0.002	− 0.04	− 0.02	− 0.03
Peru	− 0.03	− 0.01	− 0.05	− 0.03	− 0.07	0	− 0.07	− 0.08	− 0.06
Uruguay	− 0.005	− 0.002	− 0.004	− 0.003	− 0.005	0	0	− 0.001	− 0.003
Venezuela	− 0.03	− 0.02	− 0.04	− 0.02	− 0.02	− 0.003	− 0.08	− 0.07	− 0.05

Source: Harmonised Household Surveys of Latin America and the Caribbean and Oxford Stringency Index and Google Workplace Mobility Data, authors’ own calculations

Notes: *ISCO 1* refers to “Managers”, *ISCO 2* refers to “Professionals”, *ISCO 3* refers to “Technicians and associate professionals”, *ISCO 4* refers to “Clerical support workers”, *ISCO 5* refers to “Service and sales workers”, *ISCO 6* refers to “Skilled agricultural, forestry and fishery workers”, *ISCO 7* refers to “Craft and related trades workers”, *ISCO 8* refers to “Plant and machine operators, and assemblers” and *ISCO 9* refers to “Elementary occupations”. The rates for the entire LAC region have been calculated as a weighted average of the population in each country, excluding Argentina and the Bahamas, which are not representative at the national level, and Nicaragua to be consistent with the scenario under imperfect compliance. When the information was not available, we leave the cells as empty

^a^ Sample restricted to urban areas

**Table 18:** Percentage point variation in the mean loss labour income rate due to imperfect compliance, by economic activity and by country

Countries	Activity 1	Activity 2	Activity 3	Activity 4	Activity 5	Activity 6	Activity 7	Activity 8	Activity 9
All	0	− 0.01	− 0.05	0	− 0.07	− 0.04	− 0.02	− 0.02	− 0.04
Argentina ^a^	0	0	− 0.04	0	− 0.06	− 0.06	− 0.05	− 0.04	− 0.02
Bahamas ^a^									
Barbados									
Belize	0	0	0	0	0	− 0.01	− 0.004	− 0.01	− 0.02
Bolivia	− 0.007	0	− 0.03	0	0	− 0.02	− 0.04	− 0.007	− 0.01
Brazil	0	0	− 0.06	− 0.001	− 0.07	− 0.07	− 0.007	− 0.03	− 0.03
Chile	0	0	− 0.03	0	− 0.05	− 0.02	− 0.05	− 0.02	− 0.02
Colombia	0	0	− 0.02	0	− 0.04	− 0.02	0	− 0.02	− 0.02
Costa Rica	0	0	0	0	0	− 0.008	− 0.001	0	− 0.003
Dominican Republic	0	0	− 0.03	0	− 0.04	− 0.01	− 0.03	− 0.002	− 0.02
Ecuador	0	0	− 0.03	0	− 0.04	− 0.02	− 0.04	− 0.02	− 0.02
El Salvador	0	− 0.06	− 0.03	0	− 0.05	− 0.05	− 0.05	− 0.02	− 0.03
Guatemala	0	− 0.16	− 0.09	0	− 0.16	− 0.06	− 0.14	− 0.04	− 0.09
Jamaica	0	0	− 0.003	0	− 0.006	− 0.004	0	− 0.001	− 0.004
Mexico	0	− 0.06	− 0.05	0	− 0.07	− 0.01	− 0.001	0	− 0.05
Nicaragua									
Paraguay	0	0	− 0.03	0	− 0.04	− 0.02	0	− 0.03	− 0.02
Peru	0	0	− 0.06	0	− 0.08	− 0.08	− 0.08	− 0.01	− 0.03
Uruguay	0	0	0	0	0	− 0.008	0	0	− 0.003
Venezuela	0	0	− 0.06	0	− 0.09	− 0.02	− 0.08	− 0.05	− 0.03

Source: Harmonised Household Surveys of Latin America and the Caribbean and Oxford Stringency Index and Google Workplace Mobility Data, authors’ own calculations

Notes: *Activity 1* refers to “Agriculture, hunting, forestry and fishing”, *activity 2* refers to “Mining and quarrying”, *activity 3* refers to “Manufacturing industries”, *activity 4* refers to “Electricity, gas and water”, *activity 5* refers to “Construction”, *activity 6* refers to “Wholesale and retail trade”, *activity 7* refers to “Transport and storage”, *activity 8* refers to “Financial insurance and real estate” and *activity 9* refers to “Social and community services”. The rates for the entire LAC region have been calculated as a weighted average of the population in each country, excluding Argentina and the Bahamas, which are not representative at the national level, and Nicaragua to be consistent with the scenario under imperfect compliance. When the information was not available, we leave the cells as empty

^a^ Sample restricted to urban areas

**Table 19:** Percentage point variation in the mean loss labour income rate due to imperfect compliance, by individual characteristics and by country

Countries	Male	Female	Below 9 years of education	Above 9 years of education	Rural	Urban	Informal	Formal	Size firm—small	Size firm—medium	Size firm—large	Quintile total labour income—first	Quintile total labour income—second	Quintile total labour income—third	Quintile total labour income—fourth	Quintile total labour income—fifth
All	− 0.03	− 0.04	− 0.04	− 0.03	− 0.02	− 0.04	− 0.04	− 0.03	− 0.04	− 0.04	− 0.03	− 0.04	− 0.04	− 0.04	− 0.04	− 0.03
Argentina ^a^	− 0.04	− 0.04	− 0.05	− 0.03	− 0.04	− 0.10	− 0.05	− 0.03	− 0.05	− 0.04	− 0.02	− 0.06	− 0.05	− 0.05	− 0.04	− 0.03
Bahamas ^a^																
Barbados	− 0.003	− 0.002	− 0.003	− 0.002								− 0.003	− 0.003	− 0.003	− 0.002	− 0.002
Belize	− 0.007	− 0.01	− 0.009	− 0.009	− 0.007	− 0.01						− 0.01	− 0.01	− 0.01	− 0.01	− 0.01
Bolivia	− 0.02	− 0.02	− 0.02	− 0.02	− 0.01	− 0.02	− 0.02	− 0.008	− 0.02	− 0.01	− 0.007	− 0.01	− 0.02	− 0.02	− 0.01	− 0.01
Brazil	− 0.04	− 0.05	− 0.05	− 0.04	− 0.02	− 0.04	− 0.05	− 0.04	− 0.05	− 0.06	− 0.04	− 0.05	− 0.05	− 0.04	− 0.04	− 0.03
Chile	− 0.03	− 0.02	− 0.03	− 0.02	− 0.008	− 0.03	− 0.03	− 0.02	− 0.04	− 0.03	− 0.02	− 0.03	− 0.02	− 0.03	− 0.03	− 0.02
Colombia	− 0.01	− 0.02	− 0.02	− 0.02	− 0.009	− 0.02	− 0.02	− 0.01	− 0.02	− 0.02	− 0.01	− 0.02	− 0.02	− 0.02	− 0.02	− 0.01
Costa Rica	− 0.002	− 0.004	− 0.003	− 0.003	− 0.003	− 0.003	− 0.003	− 0.003	− 0.003	− 0.005	− 0.002	− 0.003	− 0.003	− 0.003	− 0.003	− 0.002
Dominican Republic	− 0.02	− 0.02	− 0.02	− 0.02	− 0.02	− 0.02	− 0.02	− 0.01	− 0.02	− 0.02	− 0.01	− 0.02	− 0.02	− 0.02	− 0.02	− 0.02
Ecuador	− 0.02	− 0.02	− 0.02	− 0.02	− 0.01	− 0.02	− 0.02	− 0.01	− 0.02	− 0.02	− 0.009	− 0.02	− 0.02	− 0.02	− 0.02	− 0.01
El Salvador	− 0.03	− 0.04	− 0.03	− 0.03	− 0.03	− 0.04	− 0.03	− 0.03	− 0.03	− 0.03	− 0.02	− 0.04	− 0.03	− 0.04	− 0.04	− 0.03
Guatemala	− 0.06	− 0.07	− 0.07	− 0.07	− 0.06	− 0.07	− 0.07	− 0.06	− 0.07	− 0.06	− 0.07	− 0.05	− 0.05	− 0.07	− 0.08	− 0.07
Jamaica	− 0.002	− 0.004	− 0.002	− 0.003	− 0.002	− 0.003			− 0.002	− 0.004	− 0.002	− 0.01	− 0.01	− 0.008	− 0.007	− 0.005
Mexico	− 0.04	− 0.03	− 0.04	− 0.03	− 0.02	− 0.04	− 0.03	− 0.03	− 0.03	− 0.03	− 0.04	− 0.03	− 0.04	− 0.04	− 0.04	− 0.03
Nicaragua																
Paraguay	− 0.02	− 0.02	− 0.02	− 0.02	− 0.01	− 0.02	− 0.02	− 0.01	− 0.02	− 0.02	− 0.01	− 0.02	− 0.02	− 0.02	− 0.02	− 0.02
Peru	− 0.05	− 0.05	− 0.04	− 0.05	− 0.02	− 0.06	− 0.05	− 0.03	− 0.06	− 0.05	− 0.02	− 0.04	− 0.05	− 0.05	− 0.05	− 0.04
Uruguay	− 0.002	− 0.003	− 0.003	− 0.003	− 0.002	− 0.003	− 0.004	− 0.002	− 0.002	− 0.004	− 0.002	− 0.004	− 0.004	− 0.003	− 0.002	− 0.002
Venezuela	− 0.05	− 0.03	− 0.05	− 0.03			− 0.05	− 0.03	− 0.05	− 0.04	− 0.02	− 0.04	− 0.04	− 0.05	− 0.04	− 0.05

Source: Harmonised Household Surveys of Latin America and the Caribbean and Oxford Stringency Index and Google Workplace Mobility Data, authors’ own calculations

Notes: The rates for the entire LAC region have been calculated as a weighted average of the population in each country, excluding Argentina and the Bahamas, which are not representative at the national level, and Nicaragua to be consistent with the scenario under imperfect compliance. When the information was not available, we leave the cells as empty

^a^ Sample restricted to urban areas

## Footnotes

1 More generally, other studies analyse the socioeconomic and health impacts of the COVID-19 pandemic (Adams-Prassl et al. 2020; Depalo 2021; Bonacini, Gallo and Patriarca 2021a; Alon et al. 2020; Baert et al. 2020; Milani 2021).

2 This constitutes a novel compilation for the LAC region. Indeed, the laws, decrees and press releases contain detailed information on (i) economic activities that are considered essential/open, (ii) activities that must cease to operate, and (iii) activities for which mobility restrictions should apply. Furthermore, the laws and decrees provide a duration for these measures. The start date is the date at which the country started being under a national lockdown. The end date was less clear because each country implemented different progressive reopening plans. Nevertheless, we can approximate the end date of the first phase of the lockdown based on the date at which there was considerable reopening of industry and/or services under certain conditions. Information on the lockdown policies implemented in LAC countries is not reported in this paper but is available in Appendix C of the GLO discussion paper (GLO DP 682 [pre]).

3 The list of countries is as follows: Argentina, the Bahamas, Barbados, Belize, Bolivia, Brazil, Chile, Colombia, Costa Rica, the Dominican Republic, Ecuador, El Salvador, Guatemala, Jamaica, Mexico, Nicaragua, Paraguay, Peru, Uruguay and Venezuela.

4 This is the case for Brazil for which we identify the measures at the state level.

5 For some countries such as Argentina, Brazil and Chile, lockdown duration also varies across regions. We use the term “region” to refer to provinces for Argentina, to states for Brazil and to communes for Chile.

6 We consider workers as living in poverty if they have a labour income below the international poverty line of $5 (2011 PPP), which was already included in the household surveys.

7 It is important to note that, in this analysis, we do not examine the health risks and related economic costs that non-compliers are facing, neither do we discuss the ethical issue that poses non-compliance. Nevertheless, we acknowledge that imperfect compliance has negative consequences on public health and poses important economic risks for societies. However, since our analysis is framed in a partial equilibrium setting and only examines the consequences of social distancing measures on labour income poverty and inequality, we find that non-compliance, by allowing a larger proportion of individuals to remain active, acts as a mechanism through which individuals smooth labour income losses.

8 Caselli (2005) uses this measure in an analysis of cross-country income differences.

9 We use data from 2019 (for some countries, earlier) in order to examine a crisis that happened in 2020. We acknowledge that using the economic structure of previous years as a baseline to measure the potential impacts of social distancing on poverty and inequality is a limitation in itself; however, in the context of an ongoing debate, this is one of the most reasonable approaches to understand the potential impacts of the current crisis.

10 In general, we expect to see some heterogeneity in the stringency of the lockdown policies applied in LAC countries, since countries in Latin America and the Caribbean have not been affected by the COVID-19 pandemic in the same proportion. According to the IADB, by the end of the year 2020, the countries that have the highest number of confirmed deaths per millions of inhabitants are Peru (1,108), Argentina (902) and Mexico (884) (IADB 2020). However, these numbers have to be taken with caution. Indeed, due to low testing, the actual extent of COVID-19 infections is highly uncertain. It is likely to be much higher in the LAC region than recorded numbers suggest.

11 If we classify occupations according to their task content into abstract, manual and routine occupations following the classification proposed by Autor, Levy and Murnane (2003) and Autor and Dorn (2013) and if we compare the corresponding employment shares that we obtain, the average shares for Bolivia and Colombia are close to the LAC average shares. If we take these employment shares as an indication that there are similarities in task content across countries in the LAC region, then this similarity would also be expressed in the teleworkability share, which is just another sphere of the task content of occupations.

12 For more information about the methodology applied to construct the 2-digit ISCO-08 variable, see Appendix B of the GLO discussion paper (GLO DP 682 [pre]).

13 This share includes workers that are working from home both fully and partially. This can explain why this share is relatively higher than the teleworking share that we have found for Chile.

14 We also rule out cases in which workers stopped to work as a precautionary measure, even when the sector remained open.

15 We also acknowledge the fact that governments implemented and/or expanded social protection programmes aimed at covering labour income falls; we discuss this later.

16 The nine sectors are the following: “Agriculture, hunting, forestry, and fishing”; “Mining and quarrying”; “Manufacturing industries”; “Electricity, gas, and water”; “Construction”; “Wholesale and retail trade, restaurants, and hotels”; “Transport and storage”; “Financial services, insurance, and real estate” and “Social, community and personal services”.

17 For a detailed explanation of the procedure, see Appendix B of the GLO discussion paper (GLO DP 682 [pre]).

18 The list of laws and decrees reviewed, as well as the estimated duration of the lockdown and the classification of the sectors, is reported specifically for each country in Appendix C of the GLO discussion paper (GLO DP 682 [pre]). Our results are in general consistent with the Oxford COVID-19 Government Response Tracker (OxCGRT) in terms of the approximate duration of the lockdowns. Concerning our definition of economic sectors at the 2-digit level, for some sectors, the definition is too general to identify some of the activities mentioned in the laws and decrees. A paramount example of this is “public transportation” which, in some countries, was closed during the lockdown. This activity appears in the ISIC Rev 4 under “Urban and suburban passenger land transport” at the 4-digit level and is part of the 2-digit category 49 “Land transport and transport via pipelines”. However, the division 49 also comprises other activities that were not closed. Since our definition is at the 2-digit level, we apply the following rule: if the proportion of workers in the class “Urban and suburban passenger land transport” was the largest compared to the other classes and “public transportation” was closed, then we assume the division 49 to be closed entirely.

19 For Brazil, we report in Fig. 2a the classification of the capital. However, in the calculations, we use a different classification across states. This information is available in Figure C.1 of Appendix C in the GLO discussion paper (GLO DP 682 [pre]).

20 The estimated duration of the lockdown varies across regions in Argentina, Brazil and Chile. For the sake of simplicity, we do not report the estimated duration for these countries in Fig. 2b. The duration of the lockdown at the regional level in these countries is available in Appendix C of the GLO discussion paper (GLO DP 682 [pre]).

21 Data on the Oxford Stringency Index can be found here: https://www.bsg.ox.ac.uk/research/research-projects/coronavirus-government-response-tracker#data. Google community mobility reports are publicly available here: https://www.google.com/covid19/mobility/. Additionally, Yeyati and Sartorio (2020b) provide an already compiled and freely accessible database that includes all the necessary information. For more information, see: https://www.utdt.edu/ver_contenido.php?id_contenido=20336&id_item_menu=32611

22 For Argentina, Brazil and Chile, the start and the end dates of the first phase of the lockdown vary across regions. Nevertheless, for the sake of simplicity, we report the dates of the capitals in Fig. 4.

23 We examine the relationship between non-compliance and some indicators of development, labour market conditions and institutionality. The results are not reported in this paper. However, it is reported in Figure A.2 of Appendix A in the GLO discussion paper (GLO DP 682 [pre]).

24 This assumption has the limitation that we are using a percentage for non-compliance that is not strictly derived from data with individuals reporting non-compliance with lockdowns as the measurement unit. However, surveys that have tried to capture this information are scarce. We still compare our proxy of non-compliance with measures obtained from surveys and find no significant difference. For instance, one survey conducted by Unicef-Argentina interviewed 2,678 households with children at the national level between the 8th and the 15th of April, 2020, and found that in 18% of the households, at least one member was not complying with the lockdown (Berho and Beccaria 2020). This percentage is close to our estimated average level of non-compliance for the city of Buenos Aires (21%).

25 We acknowledge that non-compliance might be more difficult in some sectors than others. For instance, it could be more difficult not to comply when working in closed sectors compared to working in sectors affected by mobility restrictions.

26 The Bahamas and Nicaragua are not included in this exercise since compliance in these countries cannot be computed.

27 We use the harmonised monetary total labour income available in our surveys.

28 See Appendix C Table C.2 in the GLO discussion paper (GLO DP 682 [pre]) for an approximation of the duration of the lockdown.

29 We assume that all the workers that are able to remain active receive their entire labour income. In other words, the workers do not experience any wage cuts or reduction in the number of working hours. Besides, we exclude the possibility that individuals switch to another occupation.

30 Busso et al. (2020) document a good potential coverage of the emergency social assistance programmes among the poorest households. However, there is substantial variation across countries. For instance, coverage in Chile and Ecuador reaches approximately half of the households in the first tercile, whereas in Brazil and Peru it is almost universal. Coverage is lower in the second tercile. For instance, in Colombia, Ecuador and Dominican Republic, potential coverage rates in the second tercile are below 40%. Regarding the generosity of these transfers, the replacement rate for those in the first tercile is generally high, but there are exceptions. In Bolivia, Brazil, Chile, El Salvador and Peru, the median replacement rate is 50% or more of the regular labour income. Argentina and Colombia are intermediate cases, with median replacement rates in the first tercile around 40%. The replacement rate is the lowest in the Dominican Republic and Uruguay (34 and 12%, respectively). The potential replacement rates are much lower among households in the second tercile. While the transfer exceeds 50% of the regular labour earnings for the median beneficiary household in Brazil and in El Salvador, transfers represent less than 15% of the prior and potentially forgone labour earnings of the median household in Bolivia, Colombia, the Dominican Republic, Ecuador, Peru and Uruguay.

31 Per Palomino, Rodríguez and Sebastian (2020), it also allows us to check the Pigou-Dalton transfer principle. In other words, if the labour income loss is increasing (decreasing) across the labour income distribution, then inequality falls (rises) with the lockdown for all inequality measures satisfying the Pigou–Dalton transfer principle.

32 We use the poverty line of $5 (PPP) per person per day in order to account for moderate poverty. A more extreme poverty line would not properly reflect the living conditions in Latin American and Caribbean countries. Furthermore, changes in extreme poverty are mostly explained by social transfers (Azevedo et al. 2013). Since we focus on labour income inequality, it is more appropriate to examine changes in moderate poverty.

33 For the sake of simplicity, the results for the pre-lockdown period as well as for the period at the end of the first phase of the lockdown are not reported but are available upon request. We report only the results for the changes between the two periods.

34 A similar hypothesis has been put forward in the case of informal workers in South Asia (World Bank 2020).

35 The results for Argentina and the Bahamas are not directly comparable with the results for the other countries since Argentina and the Bahamas were restricted to urban areas. Therefore, we do not include them in the explanation of the results that follows.

36 In addition, we have computed the LIC curve at the level of the LAC region (not reported in the paper). This analysis shows that the labour income loss rate is larger in the lower part of the LAC labour income distribution, where the poorest countries of the region are over-represented compared to that in the upper part of the distribution where the most developed countries are. This contributes to explain why we find an increase in inequality between countries.

37 Nicaragua’s “no lockdown” policy is left out from the exercise since it is conditional on having a lockdown. Furthermore, for the countries that have adopted a regional approach (Argentina, Brazil and Chile), we apply to all countries the lockdown policy that was implemented in the capital.

38 The value of the numerator of Eq. 7 changes according to the lockdown policy that is taken as the benchmark.

## Code availability

Available upon request.
